# Generative and Foundation-Model Artificial Intelligence in Food Safety Analysis, Authenticity Identification, and Quality Control: A Systematic Review of Validation and Decision Readiness

**DOI:** 10.3390/foods15193420

**Published:** 2026-09-24

**Authors:** Zied Bahroun, Mohamed Amine Abdeljaouad

**Affiliations:** 1 Department of Industrial Engineering, College of Engineering, American University of Sharjah, Sharjah P.O. Box 26666, United Arab Emirates; 2 CESI LINEACT, 15C Avenue Albert Einstein, 69100 Villeurbanne, France; maabdeljaouad@cesi.fr

**Keywords:** generative artificial intelligence, deep generative models, foundation models, food safety, food quality control, food authenticity, adulteration detection, synthetic data, explainable artificial intelligence, validation

## Abstract

Generative and foundation-model artificial intelligence can strengthen food analysis, but computational performance does not establish decision readiness. This systematic review examines applications and validation requirements in food safety, authenticity, and quality control. A Scopus search on 13 June 2026 identified 438 records; 65 publications were retained, including 41 direct analytical reports and 24 supporting publications. Independent dual screening and coding supported thematic synthesis and descriptive evidence mapping. The direct core comprised 18 safety-related, 4 authenticity, 9 quality-related, and 10 adjacent-domain reports. Of these, 23 reported experimental or reference-method validation, 13 dataset/model evaluation, and 5 human, sensory, or expert assessment. None of the 41 direct reports documented prospective evaluation in a real industrial setting. This finding remained unchanged in narrower-scope analyses, although the balance among experimental, dataset/model, and human-assessment validation changed. The generated food-evidence transfer gap integrates established transfer problems with food-specific decision requirements. Five cumulative gates organize provenance, technical validity, food-science validity, human calibration, and deployment governance. The framework connects generative-AI evidence requirements to established analytical standards rather than replacing them. Decision readiness requires risk-proportionate evidence for a specified use, including applicable reference-method and regulatory requirements. These findings characterize the captured literature, not the complete industrial deployment landscape.

## 1. Introduction

Artificial intelligence is increasingly embedded in food safety analysis, sample authenticity verification, quality control, nondestructive inspection, and food-property prediction. Recent advances extend beyond conventional prediction. Generative models can synthesize spectra and images, reconstruct weak signals, compensate for class imbalance, and generate synthetic or augmented data, while foundation models can integrate text, images, spectra, and domain knowledge to support interpretation and decision making. These capabilities are especially relevant where analytical evidence is scarce, heterogeneous, or costly to obtain, but they raise a harder question: when does a technically successful output become sufficiently reliable to support analytical or operational decisions?

Food safety, authenticity, and quality-control applications impose evidentiary requirements that generic artificial intelligence benchmarks do not capture. Food matrices vary by cultivar, season, batch, process, storage condition, instrument, laboratory, and production site. Safety and quality decisions draw on chemical and microbiological testing, physical and instrumental measurements, sensory and expert judgment, threshold-based risk controls, and regulatory review. A higher F1-score, visually realistic image, reconstructed spectrum, or fluent explanation therefore cannot establish practical validity on its own.

The evidence base is organized into two complementary analytical groupings: deep generative approaches emphasizing data generation and reconstruction and foundation-model approaches emphasizing interpretation and decision support. These are functional emphases, not mutually exclusive architectural classes. A large language model can itself be generative, and a broadly pretrained diffusion model can also be a foundation model. Following Bommasani et al. [1], the defining foundation-model property is broad pretraining with adaptation to multiple downstream tasks, rather than a particular generative mechanism or a universal parameter-count threshold. The two-group presentation distinguishes the evidence required for generated measurements from that required for interpretation, while allowing a hybrid system to perform both functions. Section 2.7 specifies how these groupings relate to the primary-role counts.

The distinction concerns the evidentiary role of the output. For generated spectra or images, fidelity refers to whether the output preserves physically meaningful variation and improves performance on independent real samples. For LLM-generated text, semantic coherence indicates that a statement is linguistically plausible, which does not establish factual accuracy, source support, or applicability to a particular food product or jurisdiction. A realistic Raman spectrum and a fluent account of a safety requirement therefore require different checks: chemical or microbiological correspondence in the first case and verifiable evidence and context-specific interpretation in the second [2,3,4].

Existing reviews cover large language models in food science, multimodal artificial intelligence for real-time food safety and quality, artificial intelligence in food safety, packaging, flavor analytics, nutrition, and broader food-detection technologies [5,6,7,8,9,10,11,12,13,14,15,16]. These syntheses establish the breadth of activity but leave four connected gaps. They do not consistently distinguish studies in which a generative mechanism is central from those in which it is peripheral; compare deep generative and foundation-model systems across safety and quality decisions; diagnose the validation evidence actually reported; or specify what evidence is required before model outputs can support consequential analytical or operational use.

Accordingly, this review focuses on food safety analysis, authenticity identification, and quality control, while retaining adjacent sensory, formulation, process, packaging, and nutrition applications where they clarify how validation requirements change as artificial intelligence moves from analytical screening toward downstream product and process decisions. This scope enables comparison of validation needs across analytical, interpretive, and decision-support applications without treating these adjacent domains as equally mature evidence bases.

Table 1 positions the present review against the closest prior syntheses and clarifies its distinctive emphasis on generative mechanisms, validation evidence, and the transition from model performance to reliable analytical and operational use.

This review defines the generated food-evidence transfer gap as an insufficient evidentiary connection between a generated, reconstructed, or interpreted output and its intended food-related decision. It integrates established problems of distribution shift, external validity, calibration transfer, and fit-for-purpose validation in the food context, rather than proposing a new statistical failure mechanism. Synthetic data validity concerns whether generated samples preserve real product variability; foundation-model plausibility concerns whether coherent outputs have appropriate chemical, microbiological, physical, or source-based support. The additional decision-level question is whether that evidence supports the specified action under food-matrix variation, asymmetric error costs, and applicable analytical and regulatory requirements. Classical instrument-standardization research already addresses cross-instrument transfer; the present construct incorporates rather than supersedes that knowledge [17,18].

The review is guided by four objectives:

O1. Map the structure and recent development of generative and foundation-model artificial intelligence in food safety analysis, authenticity identification, quality control, and adjacent food applications.

O2. Conduct a mechanism-based content analysis of themes, subthemes, findings, validation practices, and research priorities, with particular attention to food safety analysis, authenticity identification, and quality control.

O3. Diagnose the evidence base using transparent counts of artificial intelligence roles, food domains, and reported validation categories.

O4. Develop a validation-readiness framework that connects generated outputs to traceable, externally validated, human-calibrated, and governable use in food safety, authenticity, and quality control.

Together, these objectives structure the review around two complementary analytical groupings, a paper-traceable thematic synthesis, a descriptive validation diagnosis, and an integrative framework connecting computational outputs to analytical and operational requirements. Generative and foundation-model capabilities are examined through their contribution to the decision workflow, with domain-specific validation and governance determining the permissible use of their outputs.

The remainder of the paper is organized as follows. Section 2 describes the systematic-review design, eligibility criteria, screening and coding procedures, evidence classification, and quantitative mapping rules. Section 3 develops the mechanism-based thematic synthesis, examining analytical augmentation and reconstruction, food safety and authenticity, quality control and nondestructive inspection, adjacent sensory and formulation applications, and foundation-model and multimodal decision support. Section 4 maps the distribution of artificial-intelligence roles, application domains, validation evidence, and publication patterns across the reviewed corpus. Section 5 integrates these findings to examine the generated food-evidence transfer gap, its implications for analytical and operational use, and the validation requirements captured by the proposed readiness framework. Section 6 concludes by summarizing the main findings and identifying the conditions required for generative and foundation-model artificial intelligence to progress toward reliable use in food safety, authenticity, and quality control.

## 2. Materials and Methods

### 2.1. Review Design and Scope

The study used a systematic review design combining structured full-text extraction, evidence-tier classification, qualitative content analysis, and count-based evidence mapping. Reporting followed PRISMA 2020 [19] in Supplementary Materials. The search covered English-language journal articles and reviews published from 2016 through the search date of 13 June 2026. The direct core comprised 41 primary empirical or method-development reports. The 24 supporting publications included 1 review, Mann et al. [20], used for packaging context. Supporting publications entered the full-corpus bibliographic profile but not direct-core validation or thematic counts. Additional contextual references outside the retained set did not enter either denominator.

Scopus was selected as a multidisciplinary indexing framework for a question spanning food chemistry, microbiology, spectroscopy, sensors, engineering, and computer science. Its structured title, abstract, and keyword fields permit the same mechanism-centered query and eligibility rules to be applied across the literature, while consistent bibliographic metadata support deduplication, source mapping, and record-level traceability. Comparative research identifies Scopus as a suitable primary search system for evidence synthesis and documents its broad disciplinary reach, including technical fields relevant here [21,22]. This rationale supports a coherent source for the present validation-focused map; it does not establish that other databases contain no additional eligible studies.

The review asks how generative and foundation-model capabilities are evaluated in a defined body of food-related journal literature, rather than estimating a pooled effect or surveying all industrial implementation. The peer-reviewed journal scope was retained to provide a bibliographically stable and consistently indexed evidence base that could be screened and coded under the same rules across the food-science and computing literature. Conference papers, preprints, industrial reports, and vendor materials were outside the selected publication scope; their exclusion does not imply inferior methodological quality or an absence of relevant deployment evidence. The 2016 start year provides a broad retrieval buffer, not a proposed date of technological emergence. The exact query includes named architectures and abbreviations as well as generic terms. No systematic backward or forward citation-tracking search contributed additional records. A post hoc matched Scopus search-string sensitivity check was conducted on 10 September 2026 using the same publication-year, language, and document-type limits; Section 2.5 and Appendix G report the results. This check evaluates query formulation within Scopus and does not quantify coverage of other databases, languages, conference literature, or citation-tracking routes.

The Scopus search was executed on 13 June 2026 and returned 438 records. The exact query combined food-context, generative or foundation-model, and application blocks, together with publication-year, language, and document-type limits. Appendix F reproduces the complete strategy. Full-text availability was rechecked on 11 July 2026 before the final evidence-tier classification. The latter date concerns retrieval and eligibility assessment, not an additional database search; consequently, the 2026 publication counts represent a partial year bounded by the June search.

### 2.2. Eligibility, Evidence Tiers, and Quality Control

Eligibility required an identifiable food product, matrix, process, or food-related decision; an eligible generative, foundation-model, multimodal, or explicit synthetic-data mechanism; and a stated analytical or decision-support contribution. Safety, authenticity, and quality inspection or property prediction were the title-aligned domains. Sensory, formulation, process, packaging, and nutrition studies were retained as adjacent applications when they illustrated a food-specific validation pathway. A food label alone was insufficient: a conventional classifier without a central eligible mechanism informed the supporting tier rather than the direct core. Food-specific image-generation and representation studies were retained as methodological demonstrations, not treated as evidence of validated safety or quality decisions. Section 4.3 and Appendix G examine a narrower scope that excludes adjacent applications and two image-only demonstrations.

Direct evidence required the eligible mechanism to be central to the reported contribution, primary empirical or method-development evidence, and sufficient methodological information for descriptive coding. Supporting publications supplied conventional baselines, adjacent methods, indirect applications, or secondary context. For example, Raman-based pathogen analysis [2] provides direct analytical evidence; food-image generation without a validated product-property endpoint provides methodological rather than safety-decision evidence; and the packaging review by Mann et al. [20] supplies secondary context. Direct and supporting describe roles in this synthesis, not a universal hierarchy of study quality.

Table 2 summarizes the eligibility, evidence-tier, and validation rules applied in the review.

### 2.3. Screening and Corpus Stabilization

Both authors independently screened all 438 titles and abstracts against the predefined criteria, then independently assessed the 71 retrieved full texts, assigned evidence tiers, and documented exclusion reasons. No duplicate records were removed, so the post-deduplication and screened totals were both 438. Disagreements were adjudicated by returning to the applicable eligibility rule and the source record or full text, followed by discussion until both authors agreed. No inclusion or exclusion decision was finalized without both authors’ agreement, and no automated screening tool was used. Title/abstract screening excluded 365 records and retained 73 reports for retrieval; 2 reports were unavailable, leaving 71 for assessment; 6 failed the final domain, mechanism, or application criteria. Appendix F, Table A6, identifies these reports and their exclusion reasons. The retained set comprised 65 publications: 41 direct analytical reports and 24 supporting publications.

The study-selection process is summarized in Figure 1.

### 2.4. Data Extraction and Content-Analysis Procedure

Both authors independently extracted and coded every retained study using the same structured form. The form captured bibliographic metadata, evidence tier, technology family, primary artificial intelligence role, food domain, method, data modality, reported findings, validation basis, synthesis use, and coding rationale. Direct-core studies were also coded for paper-level focus, key finding, dominant validation challenge, and future research priority. Appendix A, Appendix B, Appendix C, Appendix D and Appendix E present the consensus direct-core coding by theme and subtheme.

Extraction focused on publication characteristics, analytical contributions, findings, and reported validation pathways. It did not provide complete study-level counts of shared dataset identities, train-on-synthetic/test-on-real (TSTR) protocols, leakage safeguards, threshold pre-specification, permutation testing, uncertainty reporting, or formal expert elicitation. These characteristics are therefore discussed as evaluation requirements rather than assigned numerical absence rates. The five human-assessment reports identify an evidence pathway; their classification alone does not establish compliance with a sensory-panel or expert-elicitation standard.

The content analysis combined deductive coding of technology, primary role, food domain, and reported validation with inductive comparison of study focus, findings, challenges, and priorities. The five themes address two complementary questions: T1 and T5 describe generation/reconstruction and interpretation mechanisms, whereas T2–T4 describe safety/authenticity, quality, and sensory/formulation application contexts. A study may inform a mechanism theme and an application theme. The themes therefore form a non-exclusive synthesis structure, not five mutually exclusive technologies or an exhaustive classification of food science. For quantitative mapping, each publication was assigned once to one primary role and one primary food domain.

After completing the independent assessments, the authors compared every eligibility, evidence-tier, primary-role, domain, validation, theme, focus, finding, challenge, and priority judgment. Any discrepancy was re-examined against the source text and the applicable predefined rule, then discussed until both authors reached agreement. The adjudication step resolved substantive differences against the evidence and established a single classification for synthesis. The final coding therefore reflects independent dual assessment followed by explicit, source-based consensus adjudication across all screening and coding domains.

Both authors appraised mechanism centrality, food-domain relevance, evidence basis, validation category, and the relationship between findings and claims using the same structured form and source-based adjudication. The resulting classifications appear in the thematic tables and Appendix A, Appendix B, Appendix C, Appendix D and Appendix E. This structured qualitative appraisal characterizes the evidence supporting each contribution; the validation categories are not study-quality scores. A formal study-level risk-of-bias instrument, composite appraisal score, and certainty-grading procedure were not applied. QUADAS-2 and PROBAST + AI provide relevant principles for diagnostic and prediction tasks, while TRIPOD + AI provides reporting guidance [23,24,25]. Their application across reconstruction, generation, and interpretive studies would require task-specific adaptation. Independent extraction using the shared form and source-based comparison was applied throughout; no separate calibration pilot was conducted, and no formal pre-consensus agreement coefficient was calculated.

### 2.5. Quantitative and Bibliographic Rules

Validation categories describe the evidence reported for a publication, not its verified freedom from bias. Dataset/model evaluation covered internal, held-out, or benchmark testing without a qualifying food-science reference, human, or operational assessment. Experimental/reference method validation required comparison with chemical, microbiological, physical, instrumental, or other food-science evidence. Human/sensory/expert-informed validation identified panel, consumer, or expert assessment. Industrial/field validation required prospective evaluation during real operation in a plant, production line, or field setting, rather than prospective data collection alone. For readiness assessment, such evaluation should specify the intended workflow, decision rules, outcomes, and monitoring in advance. A prospective laboratory study, a retrospective industrial dataset, or rapid laboratory inference alone does not demonstrate prospective industrial operation.

Each paper receives one reported validation category. Documented industrial/field evaluation takes precedence; otherwise a qualifying human, sensory, or expert assessment is recorded, followed by experimental/reference method evidence and then dataset/model evaluation. Thus, the human category can coexist with instrumental evidence within a study but is counted only once in the map. For example, Shen et al. [26] combines sensory and instrumental evidence and is assigned to the human/sensory category. This precedence identifies the reported validation pathway; it does not rank human judgment above reference measurements or convert a descriptive category into a quality or readiness score.

The counting unit is the publication, not the model, experiment, physical sample, or independent dataset. Primary-role, primary-domain, and validation maps use the 41 direct reports. Publication-year and source-title profiles use all 65 retained publications, including the supporting review. Counts describe this selected corpus, not field-wide prevalence or independent replications. Dataset reuse across publications was not systematically inventoried, so 41 reports must not be read as 41 independent datasets. No inferential statistical tests or pooled effects were calculated. The 5-paper diffusion/super-resolution group combines related analytical roles, including a GAN-based super-resolution application, rather than five exclusively diffusion-based architectures.

Exploratory within-corpus scope checks retain the original paper-level role and validation assignments while restricting the comparison set. The first restricts the direct core to its 31 title-domain reports, excluding 10 adjacent applications. The second additionally excludes the image-generation and zero-shot food-detection demonstrations of Chen et al. [27] and Zhou, P. et al. [28], yielding 29 reports. A separate bibliographic check excludes the supporting review. These checks assess sensitivity to scope and publication type; they neither add records nor estimate retrieval recall. Appendix G provides the subsets and counts.

Search-string sensitivity was examined post hoc on 10 September 2026 by rerunning the exact original query and two nested variants with identical publication-year, language, and document-type limits. The relaxed variant removed the third application block; the tightened variant retained a subset of the terms in that block. Exported records were matched by Scopus identifier, and recovery of the retained publications was checked by DOI and normalized title. The 439 records retrieved only by the relaxed formulation were then assessed conservatively against the existing scope definitions and the original 13 June 2026 evidence cut-off. Records clearly outside scope or insufficiently aligned with the mechanism and decision criteria were excluded at title/abstract stage; potentially relevant pre-cutoff records received a more detailed eligibility assessment. The sensitivity exercise was used to characterize search-boundary effects and to test the stability of the principal evidence pattern. It was not treated as a replacement systematic search because the primary corpus was defined by the locked June query and its independent dual-review workflow. Section 4.3 reports the results, and Appendix G specifies the query transformations, staged assessment, and diagnostic impact analysis.

### 2.6. Linking Objectives to Evidence and Outputs

Table 3 links each review objective to its analytical response, evidence base, and corresponding section-level outputs.

### 2.7. Technology Taxonomy

Figure 2 presents both typical functional emphases within potentially overlapping technologies. Generation and reconstruction strengthen data or representations; foundation-model applications emphasize reusable representations, interpretation, and interaction. Representation learning can support either emphasis. Broad pretraining and downstream reuse, rather than size alone, define a foundation model [1]. The combined foundation/LLM/multimodal reporting label follows the capabilities described in the publications; model scale and pretraining breadth were not independently standardized across that group.

Primary-role assignment follows the contribution evaluated as central in the publication. Generated or reconstructed measurements and features are coded by their generation/reconstruction role; foundation/LLM/multimodal contributions are coded by that principal reported capability, including its use for interpretation or data supplementation. Auxiliary components remain visible in the method description, while mechanism and application themes permit multiple interpretation contexts. A diffusion generator followed by a vision-language interpreter requires assessment of both output types even when its publication contributes once to a primary-role count.

### 2.8. Protocol, Registration, and Amendments

The review was not prospectively registered, and no separate review protocol was published. The implemented process is documented through the exact search strategy and report-level exclusions in Appendix F, the eligibility and synthesis rules in Section 2.2, Section 2.3, Section 2.4 and Section 2.5, and the paper-level thematic records in Appendix A, Appendix B, Appendix C, Appendix D and Appendix E. The July 2026 full-text availability and mechanism-centrality check preceded finalization of the 65-publication corpus and 41-paper direct core. The scope comparisons and September search-string comparison in Appendix G were exploratory post hoc analyses. This reporting makes the implemented procedure inspectable; prospective registration would additionally have provided a public record of methodological commitments before selection.

## 3. Mechanism-Based Thematic Content Analysis

The 41-paper direct core contains 18 safety/contaminant/pathogen/spoilage reports, 4 authenticity/adulteration reports, 9 quality/inspection/property-prediction reports, 8 sensory/formulation/flavor/process reports, and 2 packaging/nutrition reports. These mutually exclusive primary domains yield 31 title-domain and 10 adjacent-domain papers. The aggregate 31/41 (75.6%) describes scope composition, not superiority, maturity, or the prevalence of these applications in the wider field. Appendix G identifies all 10 adjacent reports and presents the narrower-scope counts.

Table 4 defines the five non-exclusive themes and their subthemes. Table 5, Table 6, Table 7, Table 8 and Table 9 synthesize representative evidence, findings, validation challenges, and research priorities. Paper-level tables for the complete direct core appear in Appendix A, Appendix B, Appendix C, Appendix D and Appendix E.

Theme appearances exceed 41 because the analysis operates on two complementary layers. T1 and T5 capture technological mechanisms, while T2 to T4 capture application and decision arenas. A study can inform both layers without being counted twice in the quantitative maps. Packaging and nutrition remain visible in the mechanism themes and domain map, but their evidence base is too small to justify a separate application theme.

### 3.1. Analytical Augmentation and Reconstruction

Within the direct journal corpus, generation and reconstruction principally contribute upstream analytical support rather than autonomous decision authority. They augment scarce classes, reconstruct incomplete measurements, synthesize features, and expand candidate design spaces before classification, prediction, inspection, or experimentation.

Table 5 summarizes the principal augmentation and reconstruction subthemes, findings, and validation priorities.

**Table 5:** Content synthesis for analytical augmentation and reconstruction.

Subtheme	Representative Evidence	Primary Focus	Consolidated Findings	Validation Challenge and Research Priority
Spectral and chemometric augmentation	Bao et al. [29]; He et al. [30]; Yang et al. [31]; Zhang et al. [32]; Meenakshi et al. [33]; Du et al. [2]; Sun et al. [34]; Li, X. et al. [35]	Generate or augment NIR, Raman, and resonance-Raman evidence for adulteration, toxins, pathogens, product identification, and quantitative property prediction.	Generative augmentation generally improves class separation, robustness, or quantitative prediction under small-sample conditions.	Generated spectra may not preserve chemical structure, matrix effects, instrument drift, or batch variability. Use independent batches, multiple instruments and laboratories, reference method comparison, and uncertainty reporting.
Image, feature, and signal reconstruction	Bonettini et al. [36]; Yi et al. [37]; Chen et al. [27]; Zhou, P. et al. [28]; Zhao et al. [38]; Tan et al. [39]; Williams et al. [40]; Li, B. et al. [41]	Denoise or reconstruct food X-ray, LF-NMR, terahertz, hyperspectral, and generated-image evidence, and synthesize knowledge-enhanced features.	Representation enhancement can improve downstream classification and make incomplete measurements more usable.	Visual realism, PSNR, or classifier gain is not equivalent to food-quality validity. Link reconstruction to physical properties, external samples, production conditions, response time, and cost.
Synthetic tabular, volatile, and sensory data	Ince et al. [42]; Ince et al. [43]; Wang et al. [44]; Wang et al. [45]; Sebti et al. [46]; Glumac et al. [47]	Expand spoilage, volatile-organic-compound, contamination, regulatory-exceedance, and sensory-perception evidence.	Synthetic records help stabilize learning when observations are scarce or classes are imbalanced.	Generated and observed evidence can become blurred, and results may depend strongly on the synthetic-to-real ratio. Label provenance, compare distributions, vary ratios, and evaluate on untouched real data.
Generative inverse design for packaging	Liu et al. [48]	Generate candidate material combinations for sustainable food packaging.	Inverse design can widen the search space before costly physical development.	Candidate plausibility does not establish food-contact safety, barrier performance, manufacturability, or life-cycle benefit. Build and test prototypes against established materials.

Three distinct functions emerge. Augmentation changes the training distribution by creating additional spectra, images, volatile profiles, or tabular records. Reconstruction enhances the representation available to the downstream model by improving resolution or recovering missing signal structure. Generative inverse design extends the search space by proposing candidate material combinations. These functions are often grouped under one label, but their validation burdens differ.

Across these applications, generative methods most consistently support learning when real observations are limited, imbalanced, noisy, or costly. This analytical reinforcement is particularly evident in Raman and near-infrared spectroscopy, spoilage modeling, hyperspectral analysis, autoencoder denoising, and nondestructive testing. The more difficult question is whether the generated distribution preserves the chemical, microbiological, physical, and process variability that the intended decision must tolerate.

A particular risk is overfitting to the real data’s limited natural variability. A generator trained on one batch or a stable instrument can reproduce a narrow distribution many times without representing a new cultivar, storage condition, batch, or calibration state. GAN-augmented pathogen spectra and spoilage profiles illustrate the importance of this distinction: better within-study discrimination is useful, but it does not alone demonstrate transportability to independently collected food matrices [2,33,42]. Generation should therefore remain inside training partitions, with evaluation on independent real batches and instruments.

The next step is to test transfer rather than pursue metric gain in isolation. Studies should distinguish generated from observed evidence, retain untouched real test sets, vary synthetic-to-real ratios, test independent batches and instruments, report uncertainty and calibration, and compare outputs with relevant reference methods.

The corpus supports task-specific comparisons between diffusion and GAN approaches, not a general ranking of the two families. Zhou, P. et al. [28] evaluated a region-feature diffusion model against a GAN-based feature-generation alternative within a zero-shot food-detection framework, supporting a diffusion advantage for that particular representation task. Yi et al. [37] used a denoising diffusion probabilistic model for low-field nuclear magnetic resonance image reconstruction, whereas Chen et al. [27] examined small-dataset food-image generation. Their image or representation outcomes are not directly comparable with Raman or near-infrared classification and regression outcomes reported for GAN augmentation. Moreover, the 5-study diffusion/super-resolution role includes the Real-ESRGAN-based reconstruction application of Li, B. et al. [41], so that count must not be interpreted as five exclusively diffusion-based studies. A defensible architecture comparison requires matched real-data splits, comparable training and tuning budgets, consistent downstream tasks, and food-specific validation; cross-study differences in image-quality scores or classifier accuracy do not establish universal superiority.

### 3.2. Food Safety and Authenticity as High-Stakes Validation Arenas

With 22 non-exclusive theme appearances, safety and authenticity expose the most consequential form of the evidence-transfer problem. These applications involve rare hazards, shifting prevalence, heterogeneous matrices, economic fraud, regulatory thresholds, and asymmetric error costs. A general improvement in classification accuracy is an inadequate measure of readiness.

Two decision modes can be distinguished. Sample-level systems use generated spectra, images, or profiles to improve detection of pathogens, spoilage, contaminants, residues, and adulteration. Information-level systems use language models to organize reviews, resistance evidence, or safety-related text into searchable signals. The first still depends on laboratory or analytical confirmation and the second on source provenance, expert interpretation, and escalation logic.

Table 6 summarizes the evidence and validation priorities across food safety, authenticity, contaminants, pathogens, and spoilage.

**Table 6:** Content synthesis for food safety, authenticity, contaminants, pathogens, and spoilage.

Subtheme	Representative Evidence	Primary Focus	Consolidated Findings	Validation Challenge and Research Priority
Pathogens, antimicrobial resistance, and spoilage	Du et al. [2]; Ince et al. [42]; Ince et al. [43]; Meenakshi et al. [33]; Li, Q. et al. [49]; You et al. [50]; Sun et al. [34]	Detect foodborne bacteria and Listeria, predict spoilage intensity, and support antimicrobial-resistance analysis using generative augmentation or foundation-model evidence.	Generative augmentation improves learning from scarce pathogen and spoilage evidence; foundation models broaden knowledge-assisted analysis.	Performance may shift across matrices, storage conditions, plants, sites, and hazard prevalence. Report threshold sensitivity, false-negative risk, uncertainty, external-site performance, and alignment with reference methods.
Mycotoxins, chemical contaminants, and residues	Hao et al. [51]; Williams et al. [40]; Sebti et al. [46]; Wang et al. [44]; Wang et al. [45]; Zhu et al. [52]; Zhang et al. [32]; Zhao et al. [38]; Tan et al. [39]	Predict PAHs, aflatoxins, ochratoxin, small-molecule toxins, and pesticide residues from spectral, chemical, image, IoT, synthetic-tabular, or language-model evidence.	The methods extend rare-event learning and can support earlier screening or spatial risk estimation.	Sampling representativeness and uncertainty around regulatory exceedance remain weak. Use external sampling, calibrated probabilities, regulatory thresholds, and traceable alert workflows.
Authenticity and adulteration	Bao et al. [29]; He et al. [30]; Yang et al. [31]; Shen et al. [26]	Identify milk, sweetener, medicinal-food herb, and salted-goose authenticity or adulteration patterns.	Spectral augmentation and multimodal sensory evidence strengthen discrimination under complex or limited data.	Robustness to unseen adulterants, authentic product variability, instruments, and laboratories is not established. Use unseen adulterants, independent batches, cross-laboratory validation, and standardized reference panels.
Safety risk and text analytics	Du et al. [53]; Xiong et al. [54]	Improve mushroom identification and classify food-safety reviews for surveillance.	Diffusion and LLM methods transform limited images or unstructured text into screening signals.	Text and image sources may be biased, incomplete, or misleading. Add provenance controls, expert adjudication, confidence thresholds, escalation rules, and audit trails.

These applications are especially demanding because the costs of error are asymmetric. A false negative may delay intervention, while a false positive may trigger unnecessary testing, rejection, or recall. Aggregate accuracy can obscure both risks when hazard prevalence changes across sites, products, seasons, and storage conditions.

Validation should therefore be risk-calibrated, with sensitivity and specificity reported at operational thresholds, external products and sites evaluated, unseen adulterants or hazard conditions included, uncertainty quantified, and predictions aligned with recognized reference methods. On the present evidence, these systems are best positioned as governed screening support rather than autonomous safety decision makers.

Authenticity evaluation should be anchored to the applicable chemical, isotopic, fingerprint, or sensory comparator, not discrimination accuracy alone. Reference collections, authentic variation, unseen adulterants, matrix applicability, and performance near a justified action level determine the comparison. The International Organisation of Vine and Wine (OIV) compendium provides wine-analysis methods and validation provisions, while the International Olive Council (IOC) specifies a method for virgin-olive-oil organoleptic assessment [55,56]. These references inform the food-science gate where relevant. Agreement with a reference method or official panel does not by itself establish legal acceptance of an alternative AI workflow.

### 3.3. Quality Control, Nondestructive Inspection, and Property Prediction

The quality and inspection theme is smaller but technically coherent. Its methods intervene upstream of the decision by improving or synthesizing the representation available for grading, property prediction, or classification. Its value depends on whether better representations remain useful under sensor, throughput, calibration, and process constraints.

Table 7 summarizes the principal quality-control, nondestructive-inspection, and property-prediction pathways.

**Table 7:** Content synthesis for quality, nondestructive inspection, and property prediction.

Subtheme	Representative Evidence	Primary Focus	Consolidated Findings	Validation Challenge and Research Priority
Synthetic spectra for property prediction	Zhang et al. [57]; Li, X. et al. [35]	Generate high-dimensional fruit spectra and augment near-infrared evidence for quantitative food-property prediction.	Synthetic and GAN-augmented spectra can expand the information available for fruit-quality estimation and soluble-solids prediction.	Validate spectral fidelity against measured properties across cultivars, seasons, sensors, and acquisition conditions.
Reconstruction and diffusion-based image generation	Bonettini et al. [36]; Yi et al. [37]; Chen et al. [27]; Li, B. et al. [41]	Denoise food-contaminant X-ray data, reconstruct LF-NMR and terahertz images, and generate food images from small datasets.	Denoising, reconstruction, and super-resolution make incomplete or low-quality measurements more usable.	Image-quality metrics do not establish better defect detection or internal-quality decisions. Connect reconstruction to external lots, physical outcomes, processing conditions, deployment speed, and cost.
Zero-shot and multimodal quality interpretation	Zhou, P. et al. [28]; Wu, H. et al. [58]; Yin, X.-Y. et al. [59]	Synthesize knowledge-enhanced visual features and fuse image, language, and spectral information for food-quality classification.	The methods reduce dependence on large labeled datasets and broaden interpretation across categories or time.	Use external benchmarks, temporal testing, modality ablation, and direct evaluation of downstream quality decisions.

The common contribution is improved use of weak or incomplete measurements. Synthetic spectra extend limited multispectral inputs, autoencoders and super-resolution models recover degraded measurements, and knowledge-enhanced or vision-language systems reduce dependence on large labeled datasets. These gains are better interpreted as improved observability than as evidence of improved product control.

Image realism, PSNR, SSIM, feature separability, or zero-shot accuracy may improve without stronger correspondence to internal defects, soluble solids, freshness, texture, or other physical properties. Stronger studies would link reconstruction and classification metrics to independently measured product properties and downstream decisions, using external lots, multiple sensors, temporal testing, modality ablation, and production-like acquisition conditions.

Two boundary cases are food-image generation by Chen et al. [27] and food-category representation by Zhou, P. et al. [28]. These establish food-specific computational mechanisms but not, by that fact, a validated safety endpoint or independently measured product-quality property. Their methodological contribution is distinguished from operational food analysis, and both are excluded in the narrowest scope comparison in Appendix G.

### 3.4. Adjacent Sensory, Formulation, Flavor, and Process Applications

This adjacent theme provides the most direct route from artificial intelligence output to product and process innovation. Its inclusion strengthens the safety-and-quality analysis by showing that reference method validation is not sufficient once outputs begin to influence human judgment, product formulation, and process decisions.

Table 8 summarizes the adjacent sensory, formulation, flavor, and process applications and their validation priorities.

**Table 8:** Content synthesis for sensory, formulation, flavor, and process innovation.

Subtheme	Representative Evidence	Primary Focus	Consolidated Findings	Validation Challenge and Research Priority
Recipe, formulation, and process-data support	Tkachenko et al. [60]; Bölücü et al. [61]	Generate and compare energy bar recipes and supplement food-extrusion datasets.	LLMs can widen the option space and organize early process or formulation reasoning.	Test physical prototypes and compare AI-assisted with conventional development workflows.
Sensory description and chemosensory creativity	Torrico [62]; Wang and Pellegrino [63]	Generate sensory descriptions and approximate human judgments of chemosensory creativity.	Language models can support ideation and structured comparison of sensory concepts.	Textual fluency is not sensory perception. Use trained panels, consumers, blind comparisons, uncertainty estimates, and reproducible prompting.
Astringency, taste, texture, and olfactory reasoning	He et al. [64]; Lee et al. [65]; Ma et al. [66]	Connect molecular, electronic-nose, taste, texture, and domain-knowledge signals.	Foundation models can integrate heterogeneous evidence and generate interpretable hypotheses.	Use paired chemical-sensory datasets, expert review, external products, and explicit domain constraints.
Synthetic sensory-data prediction	Glumac et al. [47]	Predict sensory-perception attributes in texture-modified plant-based smoothies from synthetic evidence.	Synthetic data supported proof-of-concept perception modeling using simulated sensory attributes.	Validate with independent panels, consumer samples, and real prototype variation.

Generative systems widen the option space by proposing recipes or candidate formulations; foundation models integrate process, molecular, sensory, and domain knowledge into hypotheses; and synthetic data methods compensate for limited panel evidence. These capabilities can accelerate ideation and experimental prioritization, although the available evidence supports decision assistance rather than autonomous product design.

Plausibility is especially easy to mistake for validity in this domain. A fluent recipe is a formulation hypothesis, not evidence that a product is physically feasible, safe, or legally marketable. For example, an LLM-proposed substitution in an energy bar may alter binding, water activity, texture, nutrient composition, or allergen exposure even when the recipe reads coherently. Evidence must therefore follow candidate formulations through prototype manufacture, instrumental and sensory assessment, process and shelf-life testing, and assessment of ingredient, additive, allergen-labeling, and claims requirements in the intended market. Codex hygiene and allergen-management guidance provide relevant risk-management principles, but applicable national requirements and their effective versions must be checked separately [67,68]. Prototype, sensory, and storage assessments of energy bars and sensory evaluation of brownies illustrate different validation steps, neither of which alone demonstrates a complete product-development or compliance pathway [60,62].

### 3.5. Foundation-Model and Multimodal Decision Support

The foundation-model theme spans safety, authenticity, quality, nutrition, sensory science, formulation, and processing. Its interpretive emphasis concerns the integration of language, knowledge, images, spectra, sensors, and domain concepts. Foundation models can also generate data, as in the extrusion data application; their placement here follows the central reported model role rather than denying that overlap. This grouping supports comparison of explanation, multimodal interpretation, hypothesis generation, and dialogue-supported decisions.

Table 9 summarizes the foundation-model and multimodal decision-support applications and associated validation priorities.

**Table 9:** Content synthesis for foundation-model and multimodal decision support.

Subtheme	Representative Evidence	Primary Focus	Consolidated Findings	Validation Challenge and Research Priority
Food-safety text and knowledge systems	Xiong et al. [54]; You et al. [50]; Zhu et al. [52]	Classify food-safety text, support antimicrobial-resistance analysis, and fine-tune language models for spectral contaminant prediction.	Foundation models can organize heterogeneous safety evidence and support knowledge-assisted screening.	Build traceable retrieval, confidence calibration, expert review, and governed escalation architectures.
Vision-language and multimodal interpretation	Ma et al. [69]; Wu, H. et al. [58]; Yin, X.-Y. et al. [59]	Integrate images, spectra, food-quality evidence, texture, taste, peptides, and nutrition signals.	Cross-modal transfer reduces dependence on single-modality evidence and opens high-throughput interpretation.	Use modality ablation, domain benchmarks, cross-product testing, and interpretable fusion.
Process, formulation, and sensory reasoning	Bölücü et al. [61]; He et al. [64]; Tkachenko et al. [60]; Wang and Pellegrino [63]; Torrico [62]; Lee et al. [65]; Ma et al. [66]	Supplement process datasets, generate formulations, interpret flavor and sensory concepts, and integrate electronic-nose knowledge.	Language models can turn fragmented data and expert language into structured hypotheses and candidate decisions.	Apply food-science constraints, expert calibration, prototypes, and comparative decision-workflow studies.

Three modes recur: text and knowledge systems that organize unstructured safety or resistance information; vision-language and multimodal systems that transfer information across images, spectra, molecular signals, texture, taste, and nutrition; and interactive reasoning systems that support formulation, process interpretation, sensory comparison, and olfactory knowledge integration.

The concern extends beyond hallucination to a broader epistemic opacity involving uncertain source provenance, hidden modality dominance, prompt sensitivity, model-version instability, weak calibration, and outputs that may appear persuasive without experimental grounding. Specifically, 4 of the 5 human, sensory, or expert-informed studies occur in the sensory, formulation, flavor, and process domain.

For these systems to function as auditable decision support, evaluation should combine domain-specific benchmarks with external product testing, retrieval and provenance controls, modality ablation, versioned prompts and models, uncertainty reporting, and expert assessment tied to a defined decision.

### 3.6. Cross-Theme Synthesis: From Generated Output to Credible Food Evidence

Taken together, the themes show an uneven evidence base. Augmentation and reconstruction are supported by larger and more experimentally anchored literature, particularly in safety, authenticity, and quality control. Foundation-model reasoning is represented by fewer studies but sits closer to interpretation and decision support. Validation burden varies by application. Safety and authenticity require stringent error control and reference-method confirmation, quality inspection requires external product and sensor transfer, and adjacent sensory and formulation uses depend more heavily on human and experimental calibration.

Table 10 summarizes the cross-theme evidence patterns, associated risks, and validation implications.

**Table 10:** Cross-theme findings, risks, and validation implications.

Cross-Theme Finding	Evidence Pattern	Risk if Ignored	Validation Implication
C1. Analytical reinforcement is the current evidence base.	Twenty-eight direct-core appearances use generative augmentation, reconstruction, diffusion, or other synthetic data mechanisms.	Internal performance may be mistaken for food-system readiness.	Require provenance, untouched real test data, external batches, and reference methods.
C2. Safety and authenticity carry the highest validation burden.	Twenty-two direct-core appearances concern pathogens, spoilage, contaminants, residues, authenticity, or adulteration.	False signals can affect public health, recalls, regulation, and trust.	Use risk-calibrated thresholds, external sites, uncertainty, and expert-governed workflows.
C3. Quality control remains a representation-to-decision challenge.	Nine studies concern quality inspection, nondestructive testing, or property prediction.	Improved image or spectral metrics may not improve grading, rejection, or process decisions.	Link technical metrics to measured properties, production conditions, and downstream decisions.
C4. Human calibration is concentrated rather than systemic.	Five direct-core studies include human, sensory, or expert-informed validation; four are in the sensory/formulation domain.	Other domains may deploy outputs without sufficient judgment calibration.	Embed food scientists, sensory experts, safety specialists, and process engineers in study design.
C5. Prospective industrial reporting is absent from the direct core.	No included direct-core report documents prospective industrial/field operation under Section 2.5.	The captured journal evidence alone cannot establish operational readiness or the absence of deployment elsewhere.	Run prospective pilots with drift monitoring, audit trails, escalation, and post-deployment review.

This unevenness underlies the generated food-evidence transfer gap. The gap can arise at several points: generated data may not preserve real variability, reconstructed signals may lose physical meaning, multimodal models may depend on unstable modality combinations, and language-model outputs may fail chemical, sensory, or operational tests. Validation requirements must therefore be matched to both the type of output and the decision it is intended to influence.

Two reporting gaps persist across the captured direct core. Human assessment is concentrated in a sensory and formulation subset, and none of the included reports document prospective industrial operation under the review definition. These observations characterize the selected literature and do not establish the absence of governed operational systems outside it.

### 3.7. Supporting Evidence and Field Perimeter

The 24 supporting publications supply conventional baselines, adjacent methods, indirect applications, and secondary context without entering the 41-paper role, domain, theme, or validation counts. They remain part of the 65-publication bibliographic profile. Table 11 accounts for all 24, including the packaging review by Mann et al. [20]. Additional reviews cited for field positioning are contextual references outside this retained set.

**Table 11:** Functions of the supporting evidence tier.

Supporting Function	Contribution to the Review	Retained Publications	Reason Not Used as Direct-Core Evidence
Predictive and analytical baselines (*n* = 13)	Conventional prediction, chemometrics, sensing, imaging, segmentation, and risk assessment provide analytical comparators.	Stasenko et al. [70]; Tempelaere et al. [71]; Guru Prasad et al. [72]; Park et al. [73]; Said and Jawale [74]; Shafique et al. [75]; Lugo-Rojas et al. [76]; Li, Q. et al. [77]; Nando et al. [78]; Pang et al. [79]; Wu, L. et al. [80]; Zhang, J. et al. [81]; Yao and Wang [82].	A central eligible generative or foundation-model mechanism was not established for the direct analytical claim.
Adjacent generative and reconstruction methods (*n* = 5)	Food-image generation, few-shot learning, and related reconstruction or classification methods define the technical boundary.	Liu et al. [83]; Li, G. et al. [84]; Zhang, C. et al. [85]; Zhang, Y. et al. [86]; Devi et al. [87].	The food-science decision link or evidence of mechanism centrality was insufficient for direct-core inclusion.
Nutrition and multimodal applications (*n* = 4)	Food assistants, nutritional assessment, image–recipe fusion, and peptide representation extend the application perimeter.	Yin, Y. et al. [88]; Cinar et al. [89]; Du, Z. et al. [90]; Purwati et al. [91].	Relevant but indirect or insufficiently connected to validated food-industry decisions.
Packaging and sustainability review (*n* = 1)	A review of natural-fiber packaging provides materials and resource-efficiency context.	Mann et al. [20].	Secondary contextual evidence; included in the full bibliographic profile but excluded from direct-core validation and theme counts.
Regulatory requirements classification (*n* = 1)	LLM classification of requirements-related provisions informs the assessment of regulatory decision-support risks.	Hassani et al. [3].	Provision classification is not equivalent to validated food-product analysis or authoritative compliance interpretation.

Contextual reviews of food processing, bioprocess engineering, and cheese manufacturing provide additional perspectives on sector-wide applications [92,93,94]. They inform field positioning without adding members to the retained 65-study corpus.

Figure 3 brings the content analysis together through the recorded theme sizes, cross-theme overlaps, and subtheme distributions. Theme bubbles represent non-exclusive appearances, and link labels report shared publications. No paper received both T1 and T5 assignments in this coding. The absent link is therefore a feature of the recorded contribution-based classification, not a claim that generation and foundation-model interpretation cannot coexist in one architecture.

## 4. Quantitative Evidence Mapping and Corpus Profile

Each of the 41 direct-core studies receives one primary assignment for the quantitative maps, while the Section 3 themes remain non-exclusive. This distinction retains cross-cutting interpretation in the qualitative synthesis without duplicating studies in the numerical results.

### 4.1. Temporal Development of the Direct Core

Figure 4 describes the publication timing of the direct core: 33 of 41 reports (80.5%) were published in 2025 or the part of 2026 covered by the search. This concentration describes the retrieved literature, not a technology-adoption rate. The absence of retained reports before 2022 does not establish absence of earlier relevant work.

### 4.2. Artificial-Intelligence Roles Across Safety, Authenticity, Quality, and Adjacent Domains

Figure 5 maps primary artificial intelligence roles across food safety, authenticity, quality control, and adjacent food domains. GAN/VAE-based augmentation and reconstruction is the largest role, represented by 19 studies, followed by foundation-model, LLM, and multimodal reasoning with 13, diffusion or super-resolution with 5, and other synthetic data generation with 4. The domain pattern reinforces the thematic findings. Augmentation dominates safety and authenticity, while foundation-model reasoning is concentrated in adjacent sensory, formulation, flavor, and process applications.

### 4.3. Reported Validation Evidence

Figure 6 records 23 experimental/reference method, 13 dataset/model, and 5 human/sensory/expert-informed reports within the 41-paper direct core. None documented prospective industrial operation under Section 2.5. This zero describes reporting within the selected journal corpus captured on 13 June 2026, not the existence of industrial systems, unpublished validation, or overall technological maturity. Human assessment is concentrated in adjacent sensory and formulation applications, whereas reference method evidence is concentrated in the title-aligned analytical domains.

The scope checks show which patterns depend on the application mix. Restriction from 41 to 31 title-domain reports yields 22 experimental/reference, 8 dataset/model, and 1 human-assessment report. Excluding the 2 image-only demonstrations further yields 22, 6, and 1 among 29 reports. All 3 subsets contain 0 documented prospective industrial reports. Thus, that bounded observation does not depend on adjacent or image-only contributions, whereas the proportions of dataset-level and human evidence do. Furthermore, 4 of the 5 human-assessment reports belong to the 10 adjacent applications and should not be generalized to safety or authenticity validation (Appendix G).

In the matched Scopus comparison conducted on 10 September, the original query retrieved 521 records, the relaxed query 960, and the tightened query 96. All 96 tightened records occurred in the original query set, and all 521 original query records occurred in the relaxed set. The original and relaxed queries recovered all 65 retained publications, whereas the tightened query recovered 22, comprising 16 direct and 6 supporting publications. Relaxation therefore added 439 records (84.3%), whereas tightening removed 425 (81.6%) and omitted 43 known included publications. Conservative assessment of the 439 relaxed-only records against the original scope and the 13 June evidence cut-off found that 419 (95.4%) did not remain in the pre-cutoff relevant set because they were excluded, conservatively borderline, or published after the cut-off. Specifically, 20 records (4.6%) remained as pre-cutoff sensitivity signals, including 5 whose characteristics were most compatible with the direct-core rules and 15 with supporting/context relevance. A diagnostic mapping of the 5 direct-core-compatible candidates preserved the 5-theme structure and the 0 count for reported prospective industrial or field validation; experimental/reference evidence would increase from 23 to 27, dataset/model evidence would remain 13, human/sensory/expert evidence would change from 5 to 6, and the title-aligned share would move from 31/41 (75.6%) to 34/46 (73.9%). These sensitivity results support a bounded interpretation: the relaxed formulation adds substantial off-scope retrieval while also revealing limited terminology-sensitive evidence beyond the locked query.

### 4.4. Evidence Orientation and Validation Depth

Figure 7 compares food domains using two directly observable proportions. The horizontal axis represents the share of foundation-model, LLM, or multimodal studies within each domain, and the vertical axis represents the share with experimental, reference method, human, sensory, or expert-informed validation. Bubble size indicates the number of direct-core studies. This design describes evidence orientation and validation depth without treating descriptive proportions as maturity scores.

The domain map describes contrasting evidence orientations. Authenticity and safety reports more often include reference evidence, while quality and inspection reports combine experimentally anchored and dataset-level evaluations. Sensory and formulation reports are more reasoning-oriented and account for 4 of the 5 human-assessment classifications. Packaging and nutrition contain only two reports. These are descriptive patterns within small, unequal groups, not estimated maturity levels or independent evidence of adoption.

### 4.5. Full-Corpus Publication Profile and Source Distribution

Figure 8 describes all 65 retained publications, including 1 supporting review. Of these, 54 (83.1%) were published in 2025 or the part of 2026 bounded by 13 June. Excluding Mann et al. [20] yields 64 publications, of which 53 (82.8%) fall in those years; the 2026 count becomes 25 rather than 26. The 41-paper primary-evidence distributions and leading source counts in Table 12 are unaffected. This comparison separates the full bibliographic profile from counts excluding secondary evidence.

## 5. Discussion

The thematic synthesis and quantitative maps converge on a common implication. Readiness depends on the conversion of generated outputs into evidence rather than on model architecture or benchmark performance alone. This section considers what that conversion requires across food-safety analysis, authenticity verification, quality control, human judgment, industrial use, and regulatory governance.

### 5.1. The Generated Food-Evidence Transfer Gap in Food Safety, Authenticity, and Quality Control

Across the reviewed reports, an important challenge is connecting generated spectra, images, synthetic records, reconstructions, and explanations to the chemical, microbiological, physical, and operational requirements of their intended use. The generated food-evidence transfer gap integrates these connections without proposing that domain shift or calibration transfer is newly discovered. Some failures concern conventional shifts between products, instruments, or sites; others concern unsupported semantic claims, uncertain synthetic data lineage, or a mismatch between demonstrated performance and authorized decision use.

This interpretation distinguishes demonstrated capability from the evidence needed for a particular action. Several analytical applications provide useful reference-linked results, while other reports remain demonstrations of representation quality or interpretation. Absence of reported prospective operation limits what this review can establish about operational readiness; it does not establish that no mature applications exist outside the corpus. The framework consequently evaluates the support required for a specified decision rather than assigning the whole technology a maturity label.

Relative to prior reviews that primarily inventory applications or focus on a single modality or domain, the present review adds a mechanism-centrality assessment that separates direct from supporting evidence; a paper-traceable synthesis centered on safety, authenticity, and quality control; and an explicit account of the evidence required for decision readiness.

### 5.2. Synthetic Data Validity Requires Food-Specific External Validation

Synthetic evidence can be useful when food samples are costly, hazards rare, labels limited, or measurements incomplete, but its value depends on distributional fidelity. A generator trained on a narrow set of batches or one instrument can reproduce those constraints and improve internal classification without capturing the variability that a new batch, laboratory, or production site will introduce. Generating additional observations does not increase the number of independently sampled real products. Apparent gains may also arise from leakage when spectra, images, or synthetic derivatives of the same source sample occur in both training and test sets. Food-specific evaluation must therefore examine matrix composition, batch variation, instrument effects, processing and storage conditions, and hazard prevalence rather than synthetic sample count alone.

Partitioning should precede generator fitting, and augmentation should be performed at the level of the physical sample and, where relevant, batch, instrument, site, or acquisition period. Replicate spectra from one specimen should not straddle training and testing, and data-dependent preprocessing and tuning should remain within training folds. TSTR (train on synthetic, test on real) evaluates one form of synthetic data utility; training on real plus synthetic data and evaluating on untouched real samples is also appropriate. Testing only on synthetic samples does not establish real-sample transfer. Kennard–Stone or SPXY partitioning can improve coverage of a measured space but does not itself ensure independent batches or sites. External testing, correlation-blocking cross-validation, and permutation tests that repeat model selection provide complementary safeguards.

Instrument transfer also has an established chemometric basis. Multivariate standardization and piecewise direct standardization relate instruments through shared measurements and transferred calibration relationships [17,18]. Generated spectra do not eliminate those requirements, and a transferred calibration still requires evaluation under the matrices and operating conditions of its intended use.

A credible minimum design includes the following:

- Separate generated from observed evidence and split by the relevant independent unit, such as product, batch, or site, before fitting preprocessing and generative models.
- Fit generators and data-dependent preprocessing only within the training partition or training fold; keep an untouched real test set and compare against strong non-generative baselines.
- Evaluate multiple synthetic-to-real ratios and report uncertainty, calibration, and performance on rare or difficult cases rather than overall accuracy alone.
- Include external products, batches, instruments, laboratories, or sites, with temporal testing where storage, seasonality, or instrument drift is relevant.
- Report sensitivity, specificity, and reference method alignment at operational safety or quality thresholds, including the consequences of false reassurance and false alarms.

### 5.3. Foundation-Model Plausibility Requires Human and Experimental Calibration

Foundation models can organize food knowledge, connect modalities, and accelerate interpretation, yet their outputs can appear authoritative even when they are chemically inconsistent, sensorially inaccurate, nutritionally unsuitable, or infeasible under operating conditions. Human-in-the-loop validation is therefore part of the technical evidence-conversion process, with its role determined by the decision being supported.

Food scientists, sensory experts, process engineers, safety specialists, quality managers, and operators play different calibration roles. Expert review can detect chemical or operational implausibility, trained panels can assess sensory correspondence, laboratory staff can confirm analytical thresholds, and process trials can reveal functionality and scale constraints. The current concentration of human validation in sensory studies is understandable, but the same principle should extend to safety alerts, quality classification, authenticity decisions, nutrition screening, and material design.

For high-consequence or ambiguous AI-driven safety alerts and authenticity verification, formal expert consultation should be triggered by predefined criteria and operate as a structured decision protocol rather than an informal plausibility check. Drawing on EFSA expert knowledge elicitation principles [95], the decision question, expert-selection criteria, evidence package, uncertainty elicitation, aggregation rule, and documentation should be specified in advance. Where feasible, experts should assess the underlying evidence independently before structured discussion, with disagreement and uncertainty recorded rather than compressed into apparent consensus. For safety alerts, the protocol should address false-negative consequences, action thresholds, confirmatory testing, escalation, and release authority. For authenticity verification, it should address matrix applicability, agreement with relevant reference methods or official panels, unseen adulterants, and whether the evidence supports screening, investigation, or a formal authenticity determination. Unresolved high-consequence disagreement or insufficient evidence should trigger confirmatory testing, abstention, or escalation, with final responsibility retained by qualified human decision makers.

Explainable-AI outputs are diagnostic aids, not independent evidence of chemical validity or causal mechanism. SHAP attributions and activation maps can describe model behavior without establishing that the relevant relationship is stable or physically meaningful. Explanations can also encourage over-reliance; human calibration should therefore test appropriate acceptance, rejection, and escalation on correct and erroneous outputs, not perceived plausibility alone [96]. An explanation does not by itself upgrade a study’s validation category.

A particularly consequential application of these concerns arises when foundation models are used for food-safety or regulatory consultation. Such use requires safeguards against both unsupported statements and correct statements applied to the wrong context. Hassani et al. [3], a supporting study on requirements-related provisions in food-safety regulations, reported average precision and recall of 89% and 87% for a fine-tuned GPT-4o configuration; a few-shot configuration increased recall to 97% but reduced precision to 65%. These results concern provision classification, not authoritative legal interpretation, and illustrate why a useful language-model capability does not eliminate consequential errors. More generally, improved aggregate benchmark performance is not sufficient evidence that a newer model will provide more dependable answers for a specific safety workflow [97,98].

A defensible workflow should retrieve evidence from an approved set of competent-authority sources and attach the jurisdiction, product category, effective date, document version, and relevant provision to each material claim. Retrieval-augmented generation can ground an answer, but retrieval alone cannot establish that the source is current, authoritative, applicable, or accurately interpreted. Source support must therefore be checked at the claim level, with obsolete provisions, missing exceptions, and conflicting requirements treated explicitly. Numerical limits, units, ingredient restrictions, and rule-based applicability checks should be handled by validated lookup or deterministic checks where possible rather than inferred from fluent prose. Unverified retrieved instructions must not override the system’s evidence and approval rules.

The system should abstain or request expert review when evidence is missing, sources conflict, applicability is ambiguous, or a safety-critical claim cannot be verified. Authorized personnel should retain responsibility for product release, rejection, recall, and compliance determinations. Expert-designed test sets should include outdated rules, incorrect jurisdictions, ambiguous products, contradictory sources, and deliberately unanswerable questions. Evaluation should report unsupported-claim rates, critical false reassurance, correct abstention, and the proportion of queries answered, rather than accuracy alone. Semantic-entropy methods can help flag unstable confabulations, but they do not establish the truth of a confidently repeated error and should supplement, not replace, source verification [4].

Model, prompt, retrieval-index, source-document, and rule versions should be logged together with the evidence retrieved, checks performed, expert decisions, and resulting actions. Material model or regulatory updates require re-evaluation against the same risk-relevant test set before continued operational use. These controls operationalize provenance, technical validity, food-science validity, human calibration, and governance without assuming that model scale, release date, or linguistic fluency certifies regulatory reliability.

### 5.4. Implications for Food Safety, Quality Control, and Industrial Implementation

For food engineering, the relevant unit of deployment evaluation is the decision workflow rather than the model in isolation. Technical success must accommodate sampling frequency, line speed, sensor availability, operator workload, calibration drift, reference confirmation, traceability, escalation, and regulatory responsibility. The selected direct-core reports do not document this complete prospective operational chain. The synthesis therefore identifies a reporting and validation gap within this evidence base, not an absence of industrial implementation across the sector.

Latency, cost, and regulatory accountability constrain different parts of this pathway, so their relative importance is architecture- and use-case-specific. A GAN used offline to augment training spectra does not necessarily add a generator to the production-line inference path, whereas online reconstruction or LLM consultation can add response-time and availability requirements. Cost also includes representative sampling, reference testing, integration, maintenance, and human review, not only computation. Across the present corpus, the most consistent implementation gap is not one factor in isolation but the absence of prospective evidence linking external performance, operational integration, and accountable decision authority. External validation and responsibility for consequential decisions therefore need to be established alongside speed and cost optimization.

A proportionate evaluation pathway begins with external performance testing, proceeds to prospective shadow-mode observation without action authority, and then examines supervised use with defined escalation and stopping rules. For example, a spoilage-screening tool should demonstrate calibrated alerts across incoming lots and storage conditions, together with confirmatory testing and clear alert ownership. Future implementation studies should assess these elements together. Autonomous safety or release decisions require evidence that the complete workflow satisfies its operational and regulatory requirements.

### 5.5. Validation-Readiness Operating Model

Figure 9 integrates the findings into an end-to-end operating architecture. Food data and domain knowledge enter a generative or foundation-model capability, and the resulting output passes through domain-specific application logic and validation layers before it can influence deployment and value creation. Human oversight, explainability, representativeness, regulatory governance, scalability, and cybersecurity operate across the pathway.

Table 13 organizes the synthesis into five cumulative evidence questions. The recurring needs in Table 5, Table 6, Table 7, Table 8, Table 9 and Table 10 connect synthetic data fidelity to provenance, model and representation evaluation to technical validity, reference-linked results to food-science validity, sensory and interpretive applications to human calibration, and operational requirements to deployment governance. Established analytical requirements provide the normative foundation, as detailed in the standards-alignment discussion below. The resulting conceptual framework supports task-specific evaluation; it is not a validated measurement scale. Technology readiness levels describe engineering development toward use, whereas the five gates examine the evidence supporting a defined claim and action. They are therefore complementary approaches to readiness assessment [99].

For allergen detection used to support product release, a false absence result can have severe consequences. Evidence should address the relevant food matrices, analytically justified detection capability, cross-contact scenarios, independent reference confirmation, and the sensitivity of the proposed decision threshold, together with clear authority for release and corrective action. The specific criteria must follow the hazard assessment and applicable requirements rather than a universal accuracy target [67,68]. For a fruit-ripeness tool used only to prioritize non-safety-critical sorting, independent measurements of ripeness, grading errors, sensor and seasonal drift, operator overrides, and workflow benefit may justify a less burdensome evaluation. The risk classification changes if the same output is used to infer safety or extend shelf life.

Risk proportionality changes the required evidence, not the need to answer all five questions. Food-science validity asks whether outputs correspond to appropriate chemical, microbiological, physical, or sensory evidence. Human and decision calibration asks whether the intended users interpret and act on those outputs appropriately. Neither gate is satisfied merely because an expert finds the output plausible. Acceptance criteria and revalidation triggers should be specified for the intended use before deployment, with unresolved high-consequence uncertainty constraining autonomy.

These gate-level requirements should also be interpreted in relation to established analytical, microbiological, sensory, and laboratory-quality standards. The five gates organize those requirements within a common evidence-conversion workflow. Table 14 makes their correspondences and generative AI extensions explicit. AOAC standard method performance requirements describe fit-for-purpose expectations; ISO/IEC 17025 addresses laboratory competence; Eurachem guides method validation; and ISO 16140-2 addresses alternative microbiological methods against a reference method [100,101,102,103]. Applicability depends on the measurand, matrix, use, and jurisdiction, including relevant amendments. The framework contributes by coordinating these established requirements across source lineage, generation, interpretation, and decision authority.

For European Union official-control use, the method hierarchy and performance requirements under Article 34 and Annex III of Regulation (EU) 2017/625 [104] must be identified at the outset. Validation or equivalence evidence must satisfy the applicable route; predictive accuracy alone does not authorize an alternative method. Legal method eligibility therefore connects food-science validity in Gate 3 with deployment governance in Gate 5. AI Act classification [105] is also use specific: Article 6 concerns the specified product/safety-component and third-party-assessment conditions or listed Annex III uses, subject to the applicable provisions. A food-safety label alone does not establish high-risk status. Where relevant requirements apply, risk management, data governance, documentation, logging, human oversight, accuracy, robustness, cybersecurity, conformity assessment, and monitoring must be addressed.

For quantitative descriptive sensory work, assessor selection and training and panel-performance evaluation should follow appropriate standards such as ISO 8586 and ISO 11132. ISO 8589 addresses test environments, ISO 4120 and ISO 5495 specific comparison designs, and ISO 13299 sensory profiles [106,107,108,109,110,111]. Consumer-acceptance and operational user studies require designs suited to their different objectives. Structured expert elicitation should specify the question, expert selection, uncertainty elicitation, aggregation, and documentation, as in EFSA guidance [95]. Expert participation and compliance with a structured elicitation protocol are distinct evidentiary properties.

Table 14 maps each readiness gate to its established basis, operational decision criterion, and generative-AI extension.

**Table 14:** Established requirements and operational interpretation of the five gates.

Gate	Established Basis	Operational Evidence and Decision Criterion	Generative-AI Extension
1. Provenance	ISO/IEC 17025; Eurachem.	Document sample/batch identity, sampling scope, reference values, preprocessing, and data rights. Lineage and applicability must be assessable.	Link synthetic outputs to source partitions, generation settings, model/prompt versions, and downstream use. Address training contamination and disclosed provenance limitations; data lineage is distinct from metrological traceability.
2. Technical validity	AOAC performance requirements; fit-for-purpose validation.	Use credible baselines and independent real tests, block correlated samples, separate tuning, and report errors and uncertainty. Meet pre-specified task criteria, not a universal accuracy cutoff.	Evaluate synthetic-to-real utility, leakage, ablations, reconstruction fidelity, model/prompt robustness, and unsupported semantic outputs.
3. Food-science validity	AOAC; Eurachem; ISO 16140-2; applicable official methods and EU 2017/625.	Compare with appropriate reference evidence across claimed matrices/ranges. Examine detection capability, bias, precision, agreement, and thresholds. Satisfy the applicable validation or equivalence route before official use.	Establish that generated signals, formulations, or interpreted claims preserve the food-specific property or meaning required for the proposed action.
4. Human calibration	Task-relevant sensory standards; commodity-specific panel methods; EFSA elicitation.	Use suitable assessors and protocols; examine performance and decision behavior. Apply task-specific criteria for agreement, reliance, override, and escalation.	Test reactions to confidently incorrect explanations, abstention, and unsupported recommendations, not fluency or perceived usefulness alone.
5. Deployment governance	Laboratory quality systems; food-safety management; applicable official-control and AI requirements.	Establish legal method eligibility, decision ownership, prospective workflow evidence, monitoring, incident response, and revalidation. Unresolved legal eligibility prevents the proposed official use.	Control source/model/prompt/index versions, monitor drift and unsupported claims, preserve logs, and reassess after material technical or operational changes.

For binary screening, predictive values should be evaluated at a plausible deployment prevalence rather than transferred uncritically from a balanced dataset. With sensitivity Se, specificity Sp, and prevalence p, PPV = Se × p/[Se × p + (1 − Sp) × (1 − p)]. In an illustrative calculation, Se = Sp = 0.95 and p = 0.01 give a positive predictive value of 16.1%; among 10,000 items, the expected counts are 95 true and 495 false positives. This is not a corpus result. It shows why threshold sensitivity, specificity, predictive values, and confirmatory action matter alongside aggregate accuracy. Continuous property prediction requires appropriate error and uncertainty measures rather than predictive values.

Where a statutory limit or justified action level applies, the decision threshold and its selection procedure should be fixed before independent evaluation and linked to that limit, matrix, and action. A cutoff optimized on the final test set does not demonstrate prospective decision performance. Uncertainty reporting should distinguish uncertainty in measured reference quantities from model-prediction uncertainty and uncertainty in performance estimates. Confidence or prediction intervals, appropriately grouped resampling, and justified conformal methods may support the latter; they do not replace metrological uncertainty or guarantee coverage under distribution shift. Reference comparisons should examine relevant bias, precision, detection capability, matrix effects, and agreement with uncertainty. Categorical agreement may include kappa alongside class-specific errors; continuous outputs require suitable agreement or concordance measures, not correlation alone [102].

Appendix H connects the gate questions to corpus examples, distinguishing reported support from requirements that remain unestablished. Its entries illustrate the interpretation of evidence rather than assign binary scores. The direct core contains no prospective industrial example meeting the review definition, so it supplies no demonstrated complete Gate 5 case. Task-specific operational criteria should be specified before evaluation, and unreported requirements should be distinguished from observed failures.

### 5.6. Research and Reporting Agenda

Further evidence syntheses should assess dataset sharing, sample-level separation, generator fitting within training folds, independent real-data testing, threshold pre-specification, uncertainty reporting, and structured human assessment through task-adapted extraction and risk-of-bias procedures. These extensions would support item-level comparisons beyond the present descriptive evidence categories. Complementary studies should evaluate the five gates prospectively across food matrices and decision risks, while broader-source updates incorporating citation tracking should adjudicate additional candidates and distinguish journal evidence from documented industrial implementation.

Table 15 summarizes the resulting research and reporting priorities.

Figure 10 condenses the evidence-conversion logic. Its four illustrated blocks group the five assessment gates in Table 13: the food-science/reference method block brings together technical validity and food-science validity, which remain distinct assessment requirements. Generated outputs must satisfy provenance, technical and domain-specific validity, human calibration, and deployment governance. Strong technical performance cannot compensate for missing reference evidence or uncontrolled operational use.

### 5.7. Limitations

The evidence cut-off is 13 June 2026; the 11 July full-text availability check did not update the identification search. The Scopus-only, English-language journal design provides a defined multidisciplinary evidence base but leaves conference papers, preprints, industrial reports, vendor materials, and potentially relevant publications in other databases or languages outside its scope. Chinese-language analytical research may therefore be underrepresented. The matched September reruns and conservative assessment of the relaxed-only increment confirm that retrieval is terminology-sensitive: 20 of 439 additional records (4.6%) remained potentially relevant within the original evidence window, while 419 (95.4%) did not remain in the pre-cut-off relevant set. Because this post hoc diagnostic did not repeat the original independent dual-review workflow across the expanded search set, it is not used to redefine the locked June corpus or to claim exhaustive recall. The unchanged query also increased from 438 records in June to 521 in September, showing that retrieval is time-dependent without separating new publication from changes in indexing or metadata. These boundaries restrict the deployment finding to the captured direct-core reports rather than the wider industrial landscape and motivate broader-source, prospectively updated evidence synthesis.

Both authors independently screened, assessed, extracted, and coded the evidence using shared predefined criteria, and every discrepancy was resolved through source-based adjudication before finalization. These procedures provide a common evidentiary basis for final decisions and make the selection and coding process traceable. A formal pre-consensus agreement coefficient was not calculated, and a separate extraction-calibration pilot was not conducted. The qualitative appraisal describes mechanism centrality and reported evidence rather than validated study-level risk-of-bias scores. Complete denominators for independent datasets, TSTR protocols, leakage safeguards, permutation tests, pre-specified thresholds, uncertainty estimates, and structured expert elicitation were not established. Consequently, those characteristics remain unquantified rather than presumed absent, limiting comparisons of methodological quality.

The review was not prospectively registered, and no separate protocol was published. Its implemented procedures are reported in Section 2.1, Section 2.2, Section 2.3, Section 2.4 and Section 2.5 and Appendix F, with paper-level traceability in Appendix A, Appendix B, Appendix C, Appendix D and Appendix E. Prospective registration would have provided a public record of methodological commitments before study selection; its absence limits assessment of departures from an advance plan. The exploratory scope and search-string comparisons in Appendix G clarify specific sensitivities but do not remove the coverage limitations. Broader-source, prospectively registered updates and task-specific framework testing are priorities for subsequent evidence synthesis.

The direct core contains 41 publications, not 41 verified independent datasets. Reuse of samples or benchmarks may make results dependent, and small subgroups are sensitive to individual papers. Because the direct core contains only 41 publications, and some subgroups contain as few as five studies, the descriptive proportions can change noticeably with the addition or removal of individual papers. Counts describe evidence composition rather than population parameters or comparative effectiveness. Publication and selective-reporting bias may favor successful models and favorable metrics; negative-result frequency was not systematically coded. Reported categories and illustrative gate examples likewise do not establish standards compliance. Empirical evaluation across defined products, users, and decisions is needed to assess the framework alongside applicable analytical validation and regulatory requirements.

## 6. Conclusions

Within the Scopus-indexed journal literature captured on 13 June 2026, generative and foundation-model applications span food safety, authenticity, quality, and adjacent food decisions. The 41 direct reports comprise 18 safety-related, 4 authenticity, 9 quality-related, and 10 adjacent-domain contributions. Their primary roles emphasize either generation and reconstruction or interpretation and decision support, without making those capabilities architecturally exclusive. Within this defined Scopus-indexed journal corpus, the absence of reported prospective industrial operation remained unchanged in the narrower-scope checks, while the balance of validation categories changed, underscoring the importance of explicit denominators.

The generated food-evidence transfer gap provides a food-specific synthesis of established transfer, validation, and decision-assurance requirements. The five gates organize how provenance, technical performance, reference-linked food-science evidence, human assessment, and governance support a specified use. Their contribution is to connect these requirements across synthetic-data and foundation-model workflows, not to replace established analytical standards or confer regulatory authorization.

For applications represented by the reviewed evidence, generated outputs are most defensibly used as bounded analytical or decision support unless the relevant operational and regulatory requirements have been demonstrated. Future evaluations should connect independent real-sample performance, pre-specified decision thresholds, uncertainty, appropriate reference methods, and prospective workflow outcomes. The reusable search and explicit evidence classifications provide a basis for extending this map as additional eligible research becomes available. The conclusions apply to the defined Scopus-indexed journal corpus and evidence cut-off; they do not address the existence or absence of industrial or commercial implementations, including proprietary or patented systems, outside the reviewed literature.

## Figures and Tables

**Figure 1:**
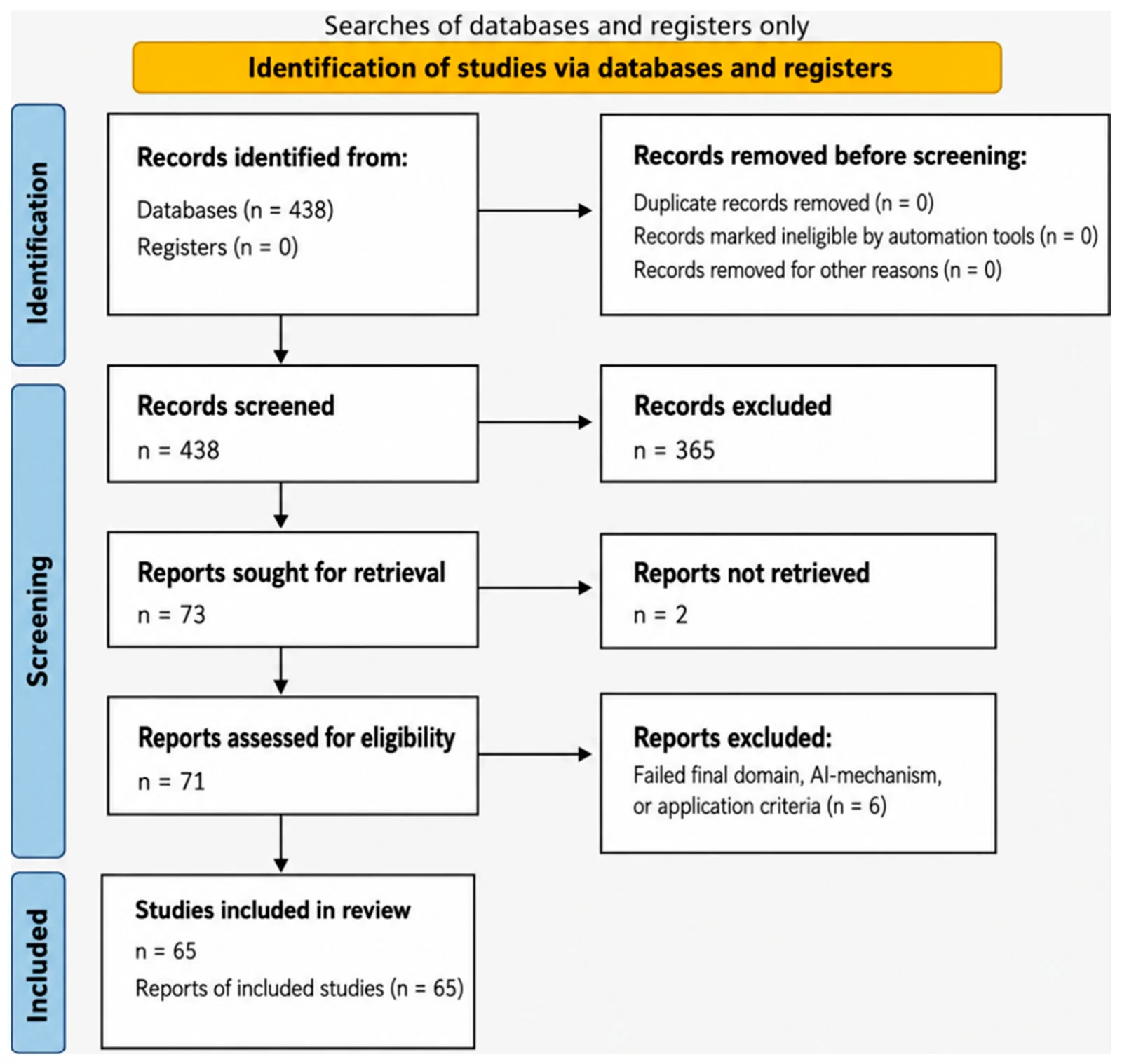
PRISMA 2020 flow diagram for the final systematic corpus.

**Figure 2:**
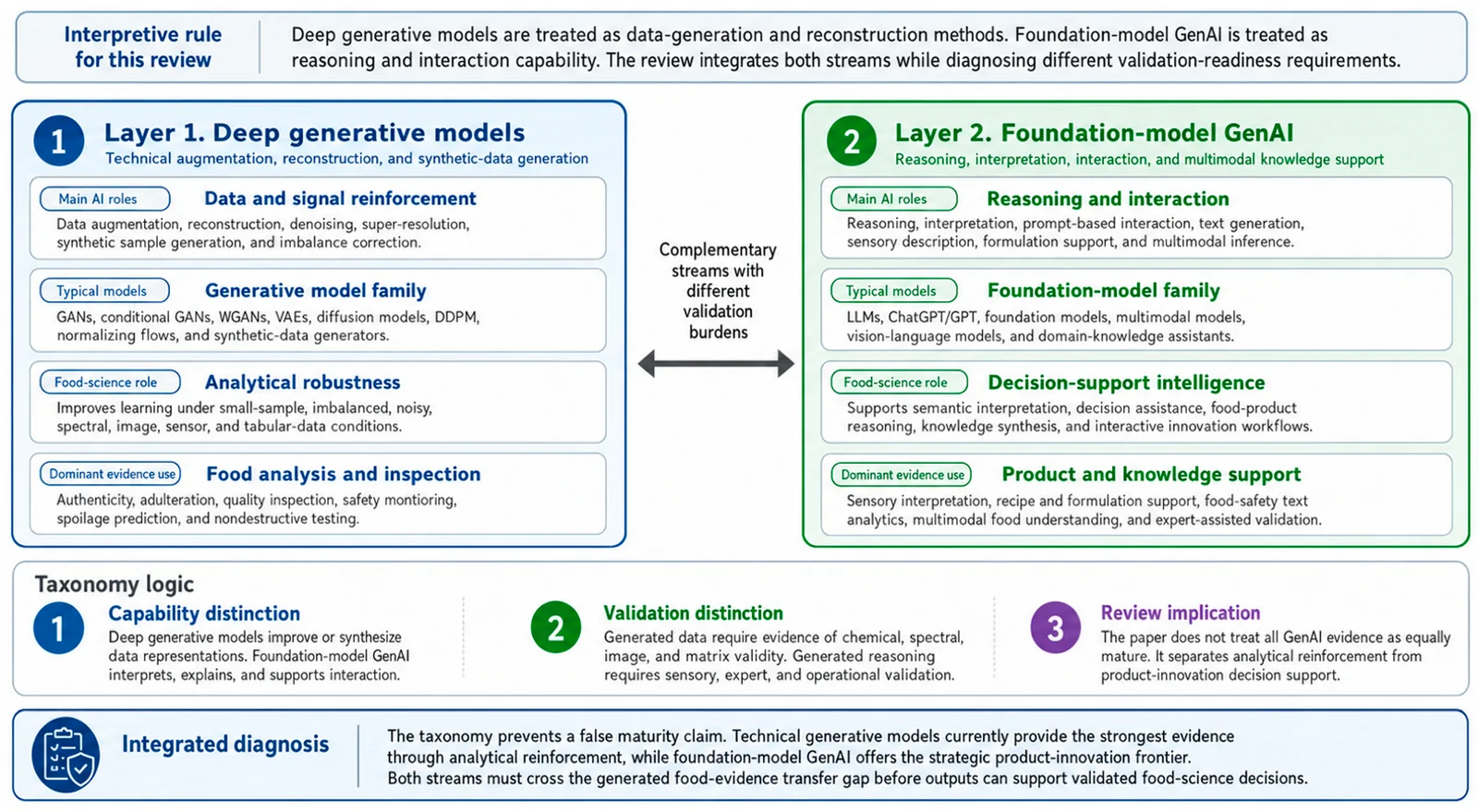
Two complementary functional emphases in generative and foundation-model food AI. Note: The groupings are not mutually exclusive architectural classes; primary-role counting follows Section 2.7. GAN, generative adversarial network; VAE, variational autoencoder; LLM, large language model.

**Figure 3:**
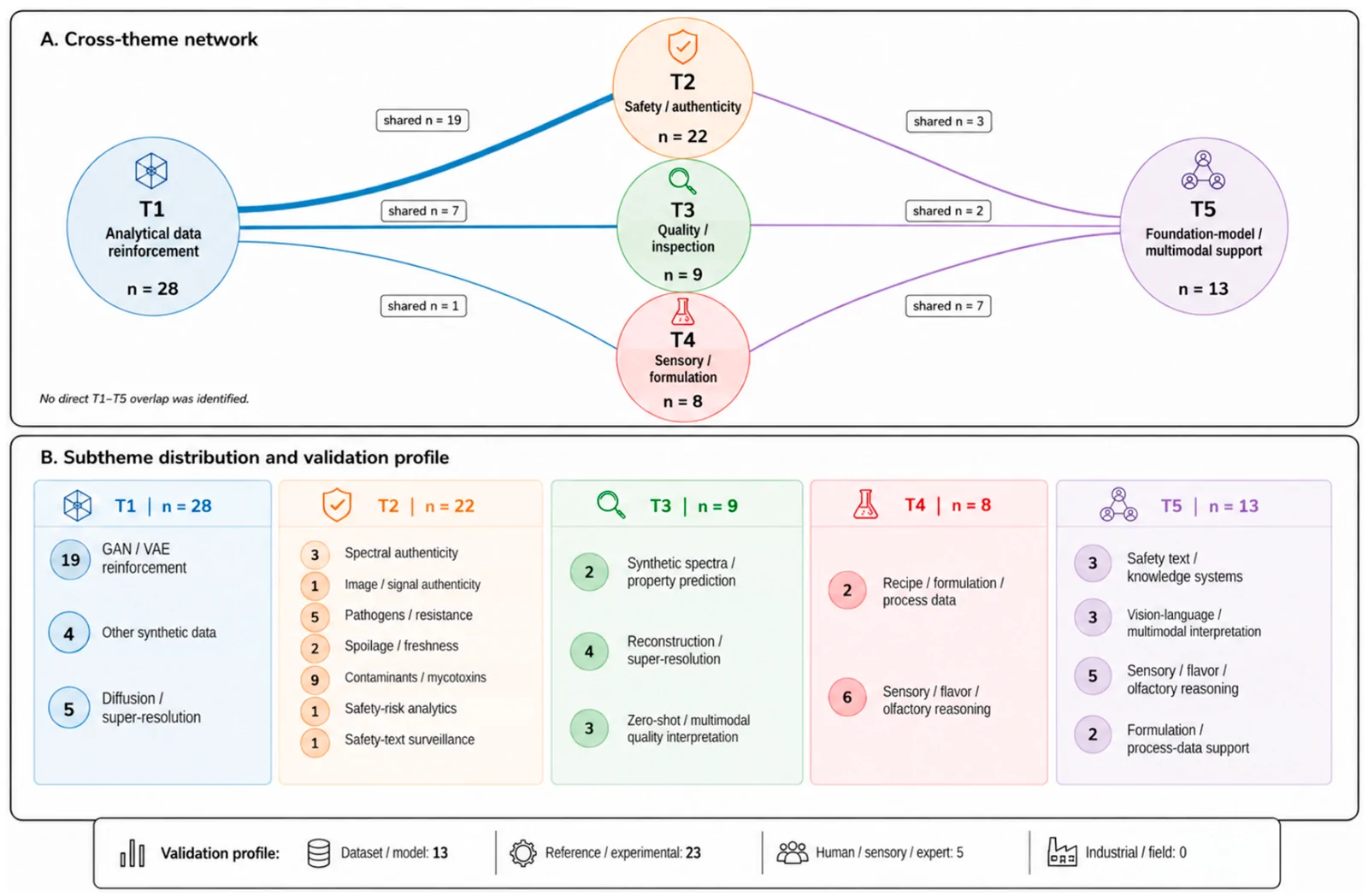
Cross-theme network, subtheme distribution, and validation profile of the 41-study direct core. Note: Theme counts and overlaps are non-exclusive; the validation counts assign each study once. The 3 T1 method groups follow the primary-role map and are not identical to the application-oriented groupings in Appendix A.

**Figure 4:**
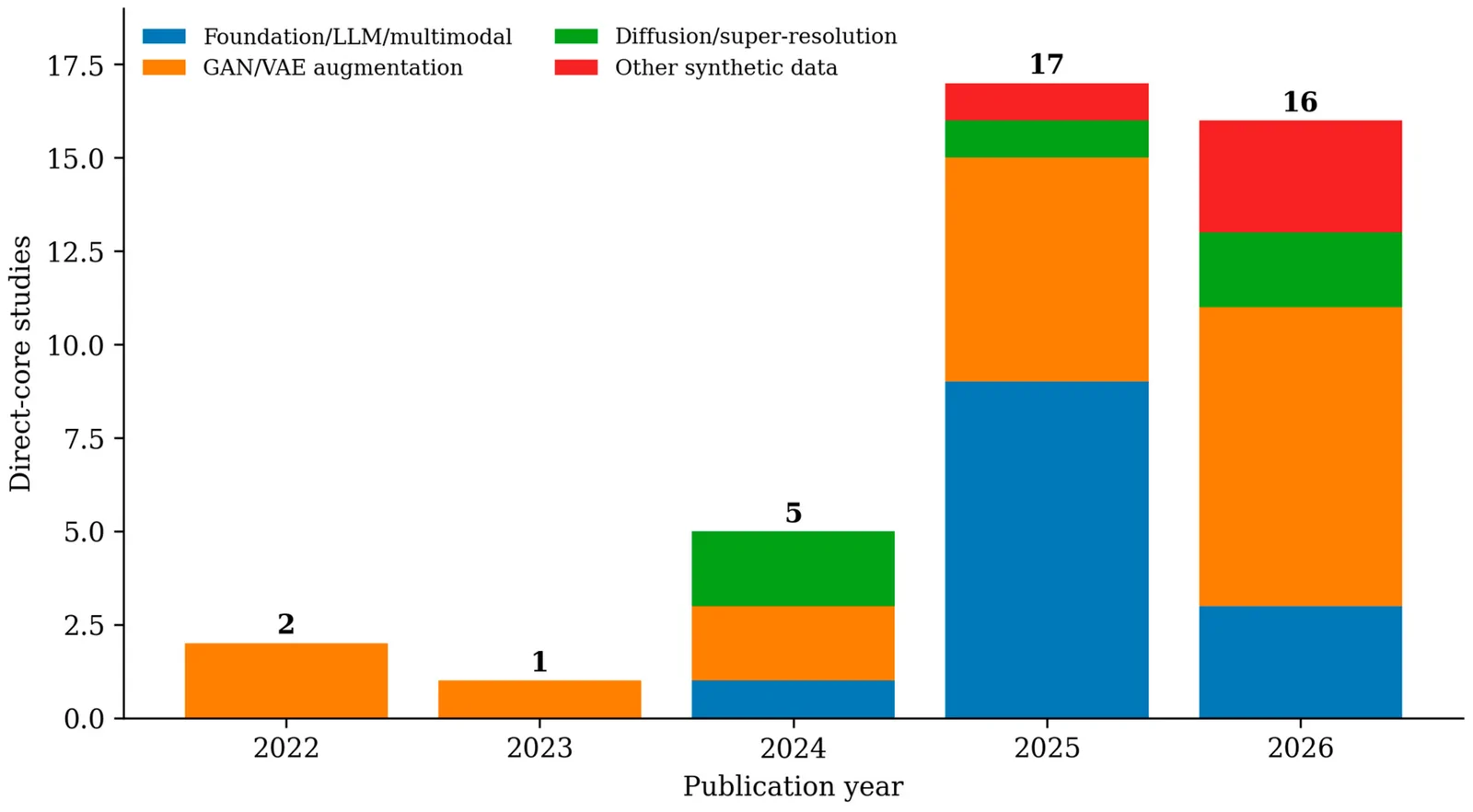
Publication-year profile of the 41 direct analytical studies.

**Figure 5:**
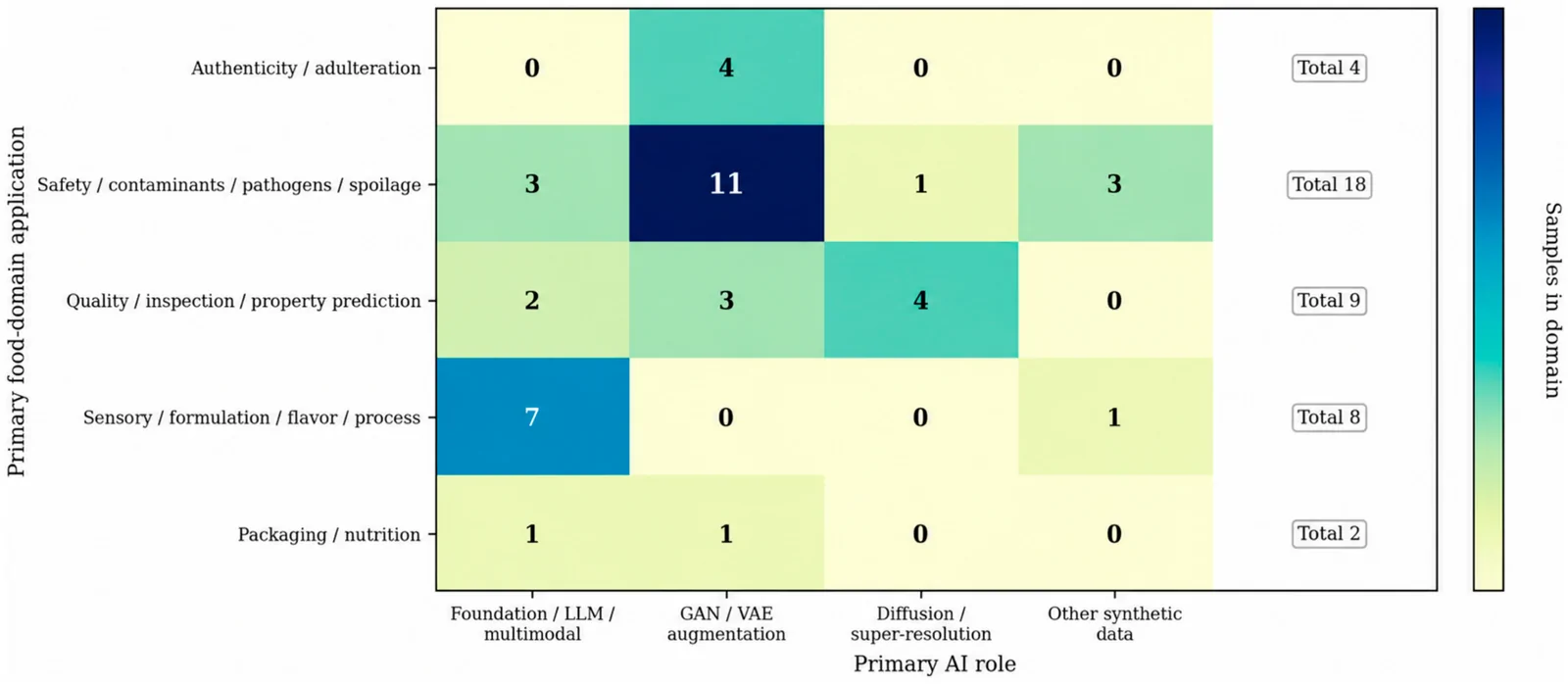
Primary artificial intelligence role across food safety, authenticity, quality control, and adjacent domains for the 41 direct analytical studies.

**Figure 6:**
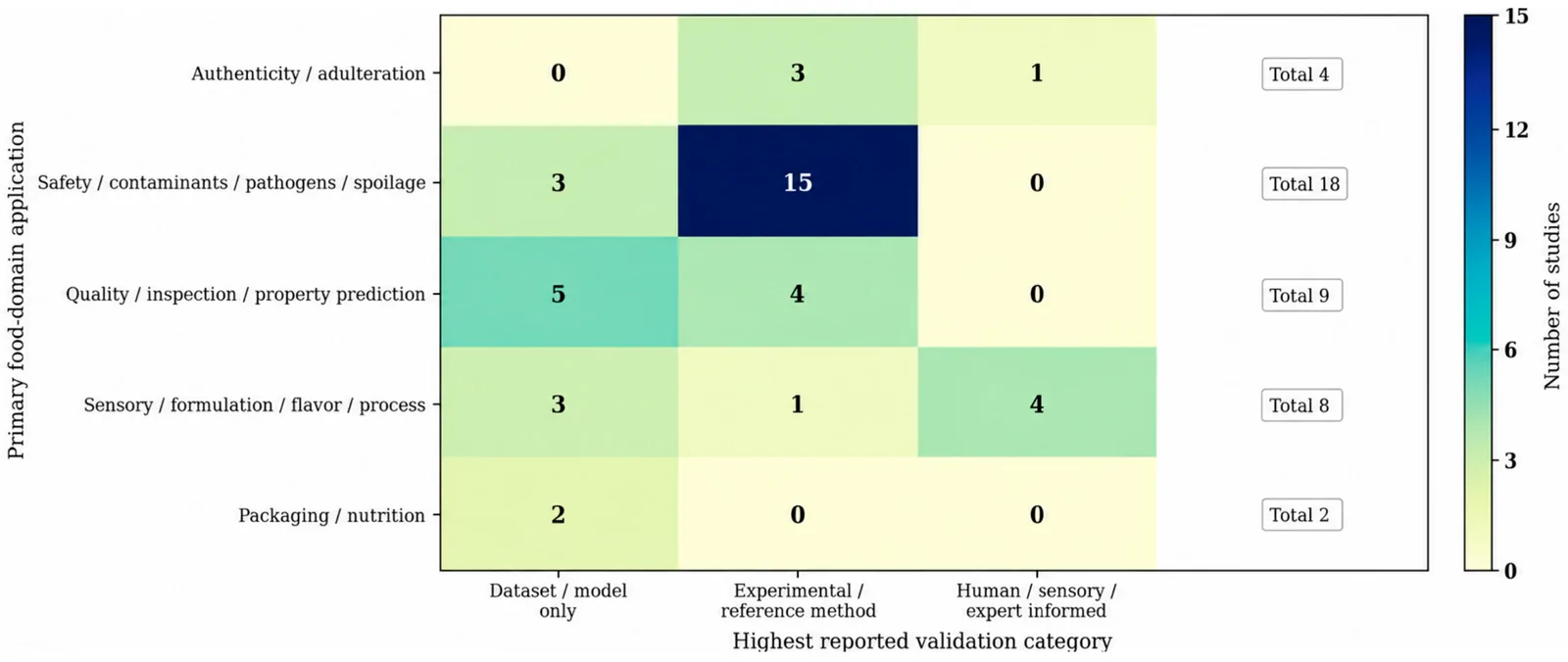
Reported validation category across food safety, authenticity, quality control, and adjacent domains in the direct-core corpus.

**Figure 7:**
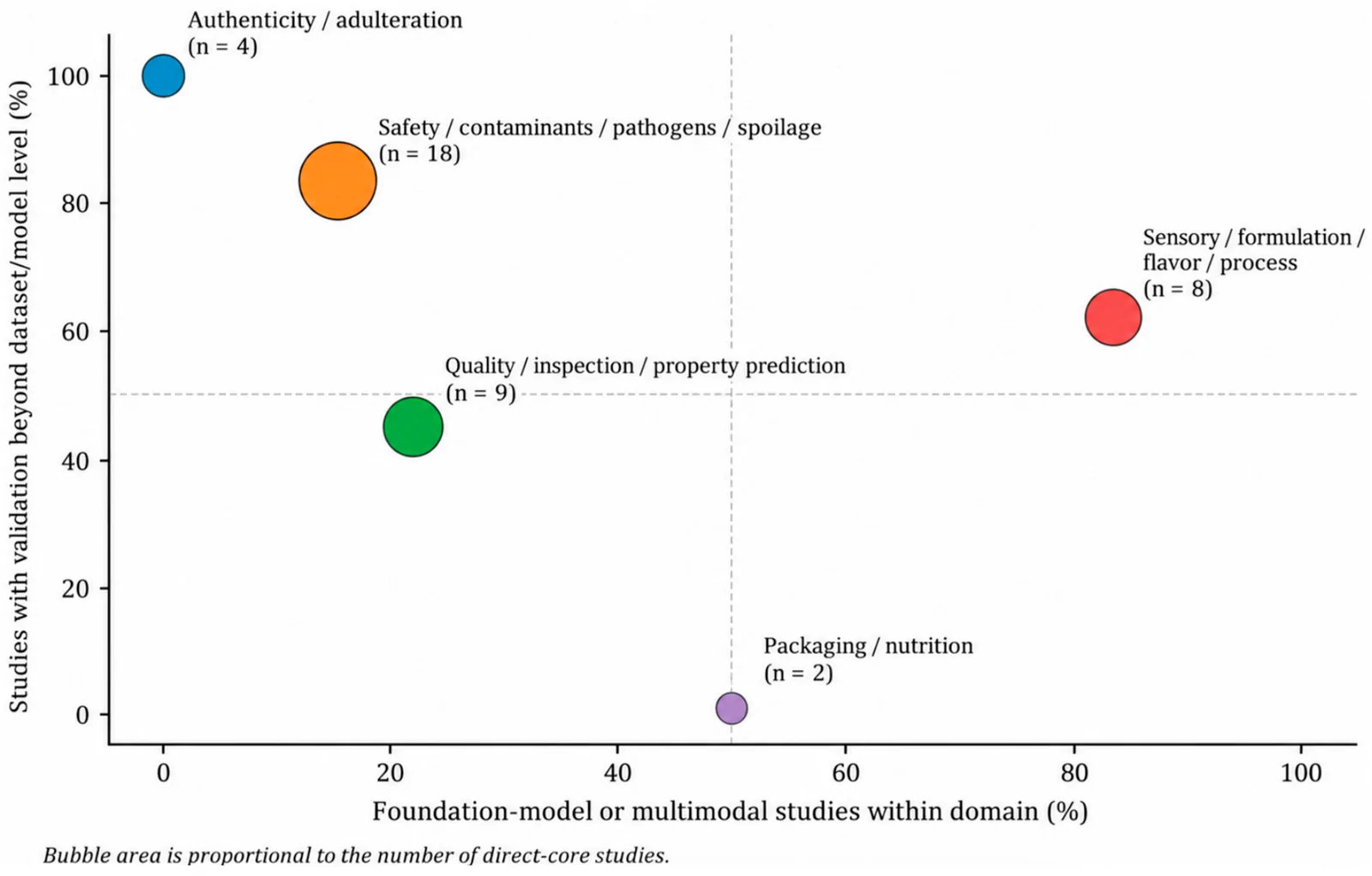
Evidence orientation and validation depth across direct-core food domains.

**Figure 8:**
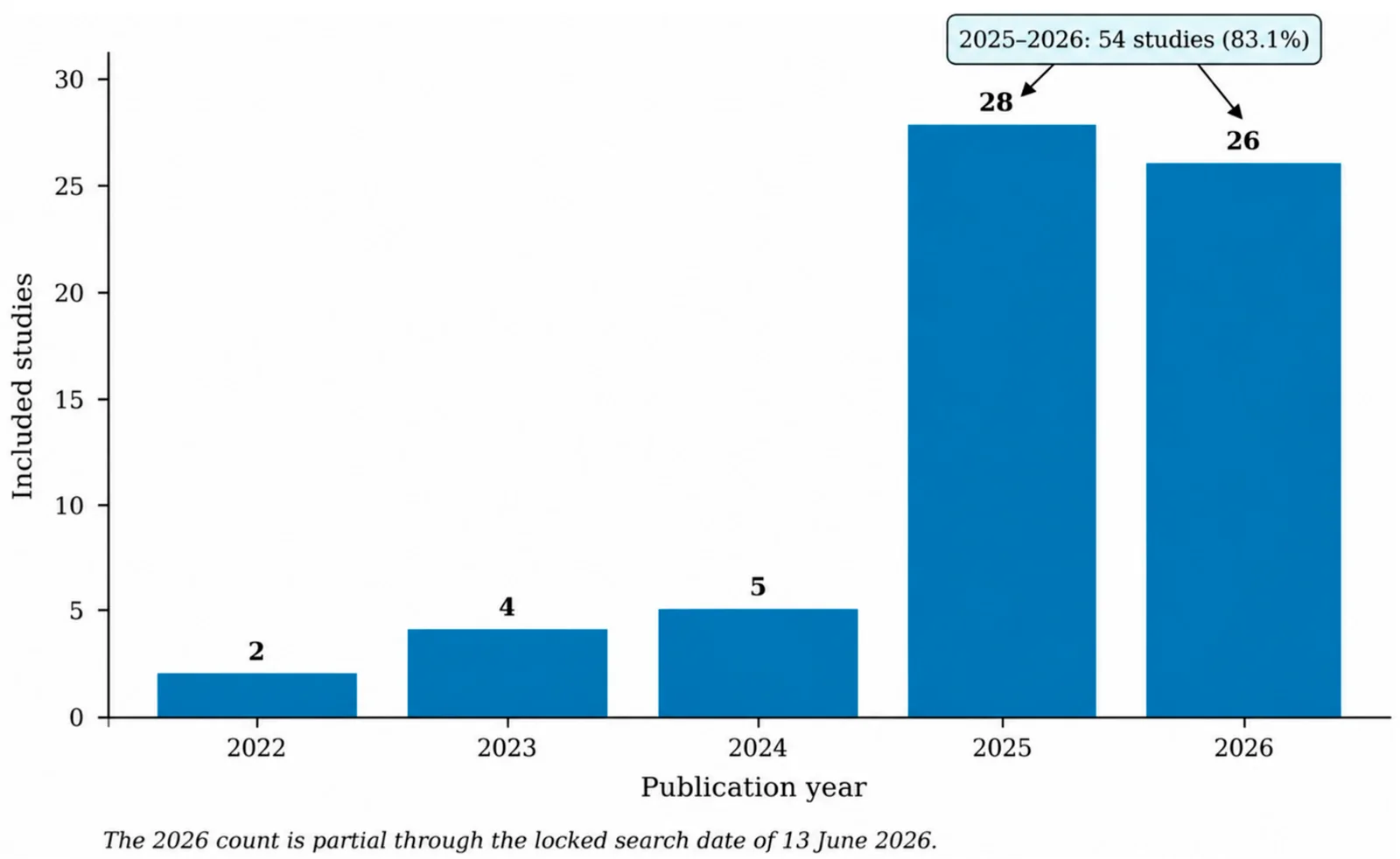
Annual distribution of the 65 retained publications, including 1 supporting review. Note: The 2026 count is partial through 13 June 2026; the review-excluded comparison is reported in Section 4.5.

**Figure 9:**
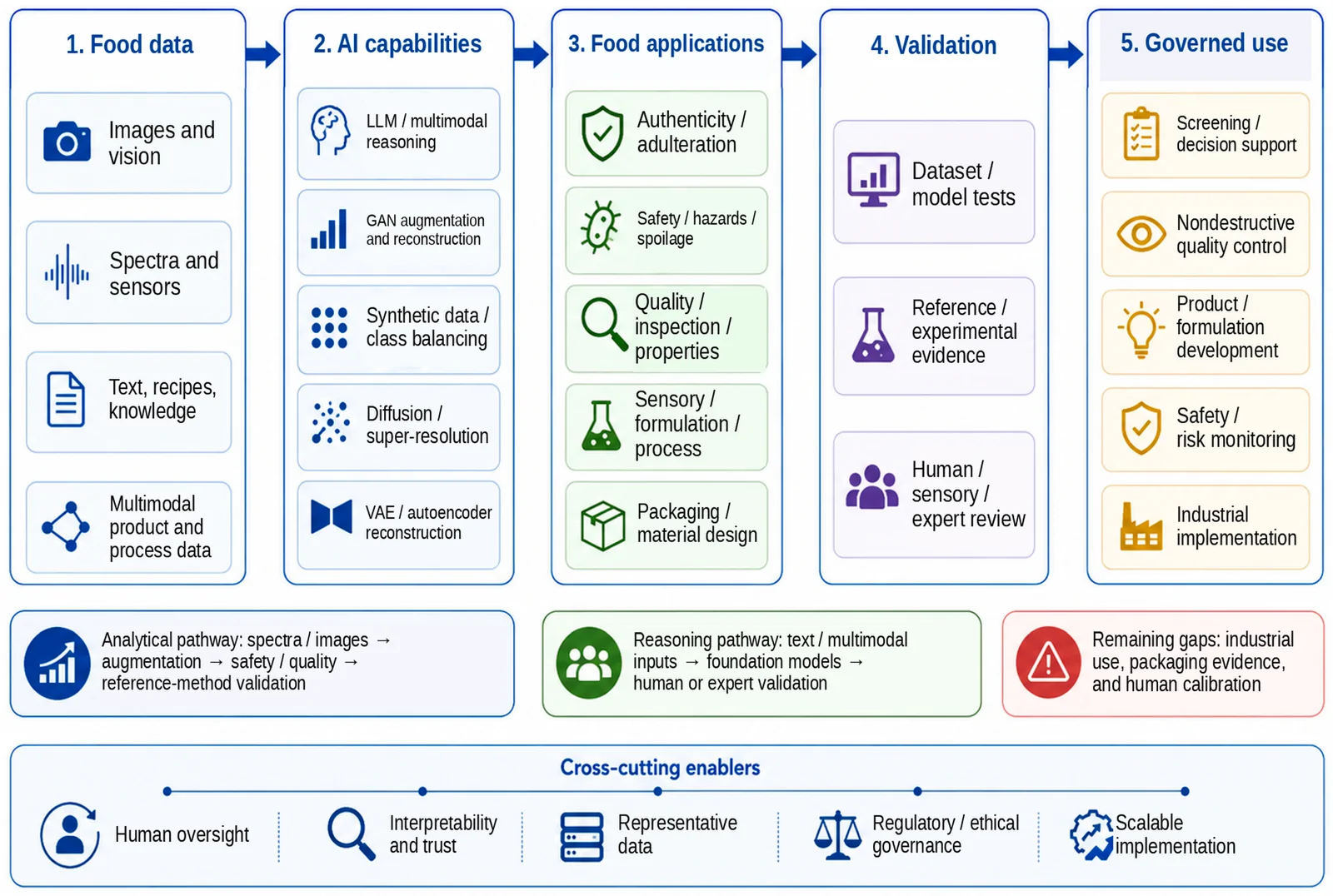
Validation-readiness operating model for generative and foundation-model artificial intelligence in food safety, authenticity, and quality control.

**Figure 10:**
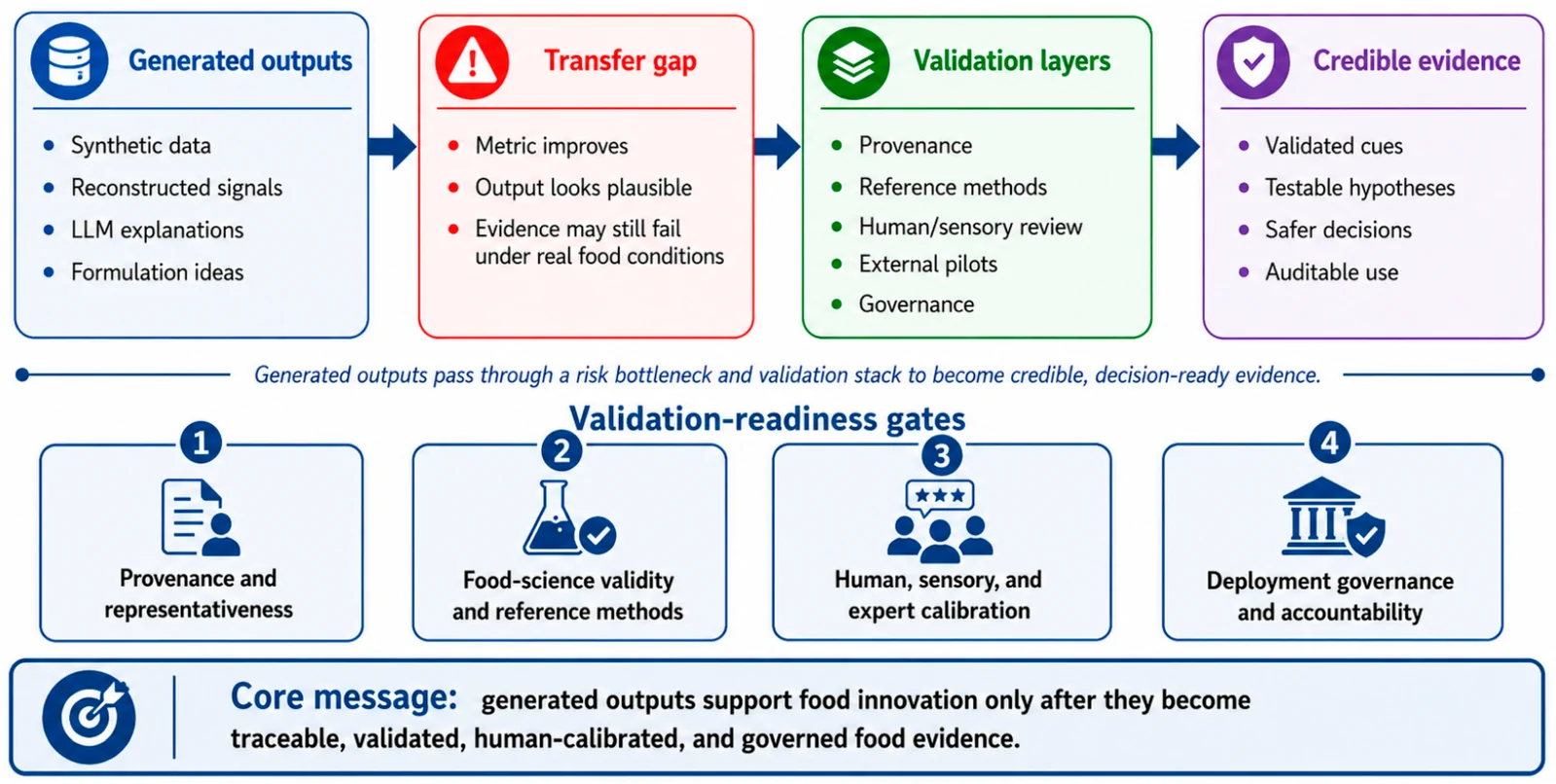
Generated food-evidence transfer gap and validation-readiness conversion logic. Note: The technical and food-science validity gates of Table 13 are grouped in the second illustrated readiness block.

**Table 1:** Positioning against the closest prior reviews. AI, artificial intelligence; LLM, large language model.

Prior Review	Primary Scope	Main Overlap	Distinct Contribution of This Review
Ma et al. [11]	Large language models in food science	Broad LLM applications and opportunities	Adds deep generative models and a validation-readiness diagnosis focused on the transfer from analytical outputs to food safety, authenticity, and quality decisions.
Chen et al. [6]	Multimodal AI for real-time food safety and quality	Sensors, foundation models, edge deployment, and regulation	Separates generative-data reinforcement from foundation-model interpretation and provides paper-level traceability of reported validation evidence.
Lopez et al. [10]	Artificial intelligence in food safety	Tertiary synthesis of food-safety AI	Focuses on generative mechanisms, evidence tiers, and the transfer from computational output to decision-ready food-science evidence.
Ding et al. [7]	Multimodal large language models for food-safety detection	Multimodal LLMs in safety-detection frameworks	Compares foundation-model reasoning with generative augmentation, reconstruction, and synthetic-data methods across safety, authenticity, and quality control.
Jia et al. [9]	AI in food packaging	Packaging technologies, challenges, and perspectives	Treats packaging as a small frontier within a broader validation architecture rather than as the primary domain.
An et al. [5]	Knowledge graphs and large language models for food safety	Knowledge-system synergy in food safety	Integrates knowledge-oriented systems with spectroscopy, imaging, contaminant detection, authenticity, and quality inspection evidence.

**Table 2:** Eligibility, evidence-tier, and validation rules.

Rule	Inclusion or Coding Logic	Exclusion or Restriction
Domain relevance	An identifiable food product, matrix, process, or food-related decision with a stated analytical or methodological contribution. Title domains and adjacent domains are distinguished in Section 2.2.	A food label alone is insufficient; methodological demonstrations do not establish validated safety or quality decisions.
AI centrality	A generative model, explicit synthetic-data mechanism, foundation model, LLM, multimodal foundation model, or generative reconstruction method is central to the contribution.	Conventional predictive AI or a generative method used only as a comparator.
Direct-core evidence	Full article available by 11 July 2026; primary empirical or method-development evidence; sufficient detail for reproducible coding.	Reviews, indirect applications, unavailable reports, or insufficiently specified mechanisms.
Supporting evidence	Comparators, adjacent methods, indirect applications, and secondary context retained for interpretation and the full bibliographic profile.	Excluded only when the study also fails food relevance or final eligibility.
Validation category	Dataset/model-only; experimental/reference method; human/sensory/expert-informed; industrial/field.	No inferred maturity score beyond what the publication reports.

**Table 3:** Objective-to-evidence alignment.

Objective	Analytical Response	Evidence Base	Main Outputs
O1. Map field structure	Publication-year profile, source distribution, technology taxonomy, and prior-review positioning.	Full 65-study corpus and direct-core metadata.	Section 2.7, Section 4.1, Section 4.2, Section 4.3, Section 4.4 and Section 4.5.
O2. Conduct content analysis	Five non-exclusive themes, subthemes, representative evidence, findings, challenges, and future priorities.	Direct core of 41 with paper-level traceability in Appendix A, Appendix B, Appendix C, Appendix D and Appendix E.	Section 3; Appendix A, Appendix B, Appendix C, Appendix D and Appendix E.
O3. Diagnose evidence	Cross-tabulations of AI role, food domain, and validation type; descriptive evidence-orientation map.	Direct core of 41, uniquely assigned by primary role and domain.	Section 4.2, Section 4.3 and Section 4.4; Appendix G.
O4. Develop readiness model	Integration of findings and validation gaps into an operating model and reporting logic.	Cross-theme synthesis plus validation evidence.	Section 5.1, Section 5.2, Section 5.3, Section 5.4, Section 5.5 and Section 5.6; Appendix H.

**Table 4:** Thematic architecture and non-exclusive direct-core evidence appearances.

Theme	Synthesis Theme	Evidence Appearances	Main Subthemes	Analytical Layer and Role
T1	Analytical augmentation and reconstruction	28	Spectral and chemometric augmentation; synthetic image and signal generation; tabular and sensor augmentation; diffusion and super-resolution.	Mechanism layer. Dominant analytical pathway.
T2	Safety and authenticity as high-stakes validation arenas	22	Pathogens and spoilage; toxins, residues, mycotoxins, and contaminants; authenticity and adulteration; safety text analytics.	Application layer. Highest validation burden.
T3	Quality, nondestructive inspection, and property prediction	9	Generated spectra and property prediction; denoising, reconstruction, and super-resolution; zero-shot and vision-language quality interpretation.	Application layer. Representation-to-decision pathway.
T4	Sensory, formulation, flavor, and process innovation	8	Recipe and formulation ideation; process-data enrichment; sensory creativity; olfactory, taste, astringency, and texture reasoning.	Application layer. Adjacent decision frontier.
T5	Foundation-model and multimodal decision support	13	Food-safety text and knowledge systems; vision-language interpretation; sensory and process reasoning; nutrition screening.	Mechanism layer. Emerging reasoning pathway.

**Table 12:** Leading source titles in the 65-publication bibliographic corpus.

Source Title	Included Studies	Interpretation
Journal of Food Composition and Analysis	6	Strong concentration in composition, authenticity, spectroscopy, and analytical quality.
Foods	4	Direct alignment with food science, sensory evaluation, data enrichment, and diffusion-based food imaging.
Food and Bioprocess Technology	3	Spoilage, processing, and data-enhanced prediction.
Food Chemistry	3	Chemical, spectral, authenticity, contaminant, and analytical validation relevance.
Sensors	3	Sensing, IoT, packaging, hyperspectral imaging, and monitoring applications.
Food Control	2	Food-safety, residue, and control applications.
Food Research International	2	Pathogen and safety-focused food research.
Computers and Electronics in Agriculture	2	Postharvest, spectral, and food-quality applications near the food-agriculture boundary.
Sensors and Materials	2	Sensor, olfactory, and quality-system applications.
IEEE Transactions on Multimedia	2	Food-image, multimodal, and food-assistant methods.

**Table 13:** Minimum validation-readiness gates for food safety, authenticity, and quality control.

Readiness Gate	Core Question	Minimum Evidence
1. Provenance and representativeness	Are the original and generated data traceable and representative of the food matrix and use context?	Document origin, sampling, food matrix, preprocessing, synthetic data labels, source versions, and representativeness for the intended use.
2. Technical validity	Does the method improve generation, reconstruction, prediction, or reasoning beyond credible baselines?	Use independent real tests, credible baselines, robustness and ablation checks, uncertainty, and metrics selected for the consequences of error.
3. Food-science validity	Do outputs align with chemical, microbiological, physical, sensory, nutritional, or process evidence?	Use relevant reference methods and defined matrix, hazard, and decision criteria. Establish the applicable validation or equivalence requirements before official-control use (see the standards-alignment discussion below).
4. Human and decision calibration	Can appropriate experts, panels, consumers, or operators interpret and validate the output in context?	Use task-appropriate assessor, panel, consumer, or operator protocols; evaluate competence, agreement, reliance, override, and escalation, not plausibility alone.
5. Deployment governance	Can the system operate safely, auditably, and robustly under industrial or regulatory conditions?	Establish legal eligibility, prospective workflow evidence, monitoring, change control, accountability, escalation, and limits on autonomy.

**Table 15:** Research and reporting agenda for validation-ready food artificial intelligence.

Priority	Current Weakness	Required Research and Reporting Shift
External and cross-matrix validation	Transfer across products, instruments, and operating settings requires evidence beyond internal model performance.	Use independent products, batches, cultivars, instruments, laboratories, plants, and time periods. Report domain shift and calibration drift.
Generated data provenance and fidelity	Synthetic-data utility depends on distinguishable provenance and fidelity to real observations.	Label synthetic data, document generation settings, evaluate distributional and chemical fidelity, and retain untouched real test sets.
Human, sensory, and expert calibration	Human validation is concentrated in a small sensory subset.	Use food scientists, trained panels, safety specialists, quality managers, and operators as part of study design, not as post hoc commentary.
End-to-end product and process studies	Validation of a proposed formulation must extend from ideation to physical and process outcomes.	Compare AI-assisted and conventional workflows using time, experimental burden, decision quality, prototype success, and acceptance outcomes.
Safety and uncertainty governance	Decision readiness requires explicit thresholds, uncertainty, false-signal costs, and escalation rules.	Report calibrated uncertainty, threshold performance, reference confirmation, alert ownership, and corrective-action closure.
Prospective industrial pilots	No direct-core publication documents qualifying prospective industrial operation; this is not a sector-wide deployment census.	Evaluate line speed, latency, reliability, monitoring, cybersecurity, auditability, role accountability, and post-deployment learning.
Sustainability and business outcomes	Resource and sustainability claims require comparison with measured baseline outcomes.	Measure samples, experiments, time, energy, waste, development cost, and commercial decision outcomes against a credible baseline.

## Data Availability

The evidence reported for the retained corpus is presented within the article and Appendix A, Appendix B, Appendix C, Appendix D, Appendix E, Appendix F, Appendix G and Appendix H, including paper-level thematic records, the original search strategy and sensitivity-query transformations, full-text exclusions, aggregate sensitivity counts, the adjacent-domain inventory, and illustrative gate-to-evidence mapping.

## Supplementary Materials

The following supporting information can be downloaded at: https://www.mdpi.com/article/10.3390/foods15193420/s1, File S1: PRISMA 2020 checklist [19] accompanies the submission. The exact search strategy and report-level exclusion reasons are included in Appendix F of this article.

## Appendix A. T1 Analytical Augmentation and Reconstruction

Table A1 provides paper-level traceability for Theme T1.

**Table A1:** Paper-level traceability for T1. Theme evidence appearances: *n* = 28.

Subtheme	Study	Study Focus	Principal Finding	Method or Mechanism	Reported Validation
A1. GAN/VAE and related augmentation or reconstruction	Bao et al. [29], camel milk adulteration	Generative and explainable NIR adulteration detection.	Improved discrimination under limited spectral evidence.	U-Mamba-Spectra; generative NIR learning.	Experimental/reference method
A1. GAN/VAE and related augmentation or reconstruction	Bonettini et al. [36], hyperspectral X-ray denoising	Denoise hyperspectral X-ray measurements for food inspection.	Autoencoder reconstruction improved usable signal quality in real time.	Autoencoder variants.	Dataset/model-only
A1. GAN/VAE and related augmentation or reconstruction	Du et al. [2], foodborne bacteria	Detect foodborne pathogenic bacteria from Raman spectra.	Adversarial augmentation improved small-sample classification.	GAN plus SVM.	Experimental/reference method
A1. GAN/VAE and related augmentation or reconstruction	Hao et al. [51], PAHs in roasted lamb	Analyze polycyclic aromatic hydrocarbons from spectral and chemical evidence.	Data-enhanced learning improved contaminant estimation.	Machine learning with generative/data augmentation.	Experimental/reference method
A1. GAN/VAE and related augmentation or reconstruction	He et al. [30], sweetener adulteration	Detect synergistic sweetener adulteration using Raman spectroscopy.	CVAE augmentation improved discrimination and feature robustness.	ResAttnCVAE and gated SpecFormer.	Experimental/reference method
A1. GAN/VAE and related augmentation or reconstruction	Ince et al. [42], sausage spoilage	Predict sausage spoilage intensity under limited observations.	TVAE augmentation improved prediction, while SHAP/LIME supported interpretation.	Tabular variational autoencoder (TVAE) augmentation plus explainable ML.	Experimental/reference method
A1. GAN/VAE and related augmentation or reconstruction	Ince et al. [43], VOC-based sausage spoilage	Predict spoilage from volatile-organic-compound profiles.	TVAE augmentation supported VOC-based spoilage prediction.	Tabular variational autoencoder (TVAE) augmentation plus explainable ML.	Experimental/reference method
A1. GAN/VAE and related augmentation or reconstruction	Li, Q. et al. [49], single-cell pathogens	Identify foodborne pathogens from Raman evidence.	GAN-enhanced Raman spectrograms improved single-cell classification.	Raman Mel spectrograms plus GAN.	Experimental/reference method
A1. GAN/VAE and related augmentation or reconstruction	Li, X. et al. [35], watermelon soluble solids	Predict soluble-solids content from NIR spectra.	Auxiliary regression GAN augmentation improved quantitative prediction.	Regression GAN plus chemometrics.	Experimental/reference method
A1. GAN/VAE and related augmentation or reconstruction	Meenakshi et al. [33], Listeria in Queso fresco	Detect Listeria using NIR spectral analysis.	GAN-augmented spectra improved detection in a food matrix.	GAN plus NIR analysis.	Experimental/reference method
A1. GAN/VAE and related augmentation or reconstruction	Shen et al. [26], salted-goose breed discrimination	Discriminate salted-goose breeds using sensory and instrumental evidence.	GAN-integrated learning improved breed authentication.	Chemometrics, sensory evaluation, GAN framework.	Human/sensory/expert-informed
A1. GAN/VAE and related augmentation or reconstruction	Sun et al. [34], limited-data pathogen identification	Identify foodborne pathogens from small Raman datasets.	Peak-aware attention improved limited data identification.	Raman attention model with synthetic data support.	Experimental/reference method
A1. GAN/VAE and related augmentation or reconstruction	Tan et al. [39], Hami-melon pesticide residues	Identify pesticide residues from hyperspectral images.	DCGAN augmentation improved residue classification.	Improved DCGAN plus hyperspectral imaging.	Experimental/reference method
A1. GAN/VAE and related augmentation or reconstruction	Williams et al. [40], aflatoxin in pistachios	Estimate aflatoxin infection from hyperspectral images.	Data-enhanced hyperspectral learning supported contamination-level estimation.	Hyperspectral imaging plus machine learning.	Experimental/reference method
A1. GAN/VAE and related augmentation or reconstruction	Yang et al. [31], medicine–food homologous herbs	Identify medicine–food homologous herbs from NIR spectra.	NIR-GAN augmentation improved small-sample identification.	NIR-GAN.	Experimental/reference method
A1. GAN/VAE and related augmentation or reconstruction	Zhang et al. [57], fruit spectral generation	Generate hundreds of wavelengths from limited multispectral inputs.	Generated spectra supported downstream fruit-quality applications.	Deep spectral generation.	Experimental/reference method
A1. GAN/VAE and related augmentation or reconstruction	Zhang et al. [32], small-molecule toxins	Identify toxins through ultraviolet resonance Raman evidence.	Frequency-consistent augmentation improved toxin identification.	Generative Raman augmentation.	Experimental/reference method
A1. GAN/VAE and related augmentation or reconstruction	Zhao et al. [38], apple pesticide residues	Detect apple pesticide residues from FT-NIR spectra.	GAN reconstruction and reverse feature fusion improved detection.	CNN-BiGRU self-attention plus GAN reconstruction.	Experimental/reference method
A2. Diffusion, super-resolution, and generative feature synthesis	Chen et al. [27], small-data food image generation	Generate single-category food images under small-data conditions.	The improved diffusion model increased visual diversity and classifier support.	Improved diffusion model.	Dataset/model-only
A2. Diffusion, super-resolution, and generative feature synthesis	Du et al. [53], mushroom identification	Generate complex-environment mushroom images for food-safety identification.	Synthetic augmentation improved identification under class and scene variability.	Diffusion/synthetic-image augmentation.	Dataset/model-only
A2. Diffusion, super-resolution, and generative feature synthesis	Li, B. et al. [41], pumpkin-seed plumpness	Detect pumpkin-seed plumpness from terahertz images.	Compressed sensing and super-resolution improved internal-quality visibility.	Terahertz compressed sensing plus Real-ESRGAN super-resolution.	Experimental/reference method
A2. Diffusion, super-resolution, and generative feature synthesis	Yi et al. [37], LF-NMR fruit imaging	Reconstruct low-field NMR images for nondestructive testing.	DDPM super-resolution improved image usability.	Denoising diffusion probabilistic model.	Experimental/reference method
A2. Diffusion, super-resolution, and generative feature synthesis	Zhou, P. et al. [28], zero-shot food detection	Synthesize knowledge-enhanced visual features for unseen food classes.	Feature synthesis improved real-world zero-shot detection.	Region-feature diffusion model for knowledge-enhanced feature synthesis.	Dataset/model-only
A3. Synthetic tabular, sensory, and spatiotemporal data	Glumac et al. [47], smoothie sensory perception	Predict sensory attributes in texture-modified plant-based smoothies.	Literature-informed synthetic data supported prediction of simulated sensory attributes.	Synthetic data plus deep neural networks.	Dataset/model-only
A3. Synthetic tabular, sensory, and spatiotemporal data	Sebti et al. [46], ochratoxin A in grapes	Classify ochratoxin contamination in real time.	AI-enhanced sensing supported rapid contamination screening.	IoT sensing with data enhancement.	Experimental/reference method
A3. Synthetic tabular, sensory, and spatiotemporal data	Wang et al. [44], Aflatoxin M1 prediction	Predict Aflatoxin M1 under distributed data conditions.	Federated learning and data enhancement improved prediction.	Federated learning with data enhancement.	Experimental/reference method
A3. Synthetic tabular, sensory, and spatiotemporal data	Wang et al. [45], spatiotemporal Aflatoxin M1 risk	Predict raw-milk Aflatoxin M1 over space and time.	Spatiotemporal attention improved risk forecasting.	STANet and synthetic/data-enhanced evidence.	Experimental/reference method
A4. Generative inverse design for packaging	Liu et al. [48], sustainable packaging inverse design	Generate candidate food-packaging materials.	Inverse design widened the material search space.	Sensor-integrated GAN inverse design.	Dataset/model-only

## Appendix B. T2 Safety and Authenticity as High-Stakes Validation Arenas

Table A2 provides paper-level traceability for Theme T2.

**Table A2:** Paper-level traceability for T2. Theme evidence appearances: *n* = 22.

Subtheme	Study	Study Focus	Principal Finding	Method or Mechanism	Reported Validation
B1. Pathogens, antimicrobial resistance, and spoilage	Du et al. [2], foodborne bacteria	Detect foodborne pathogenic bacteria from Raman spectra.	Adversarial augmentation improved small-sample classification.	GAN plus SVM.	Experimental/reference method
B1. Pathogens, antimicrobial resistance, and spoilage	Ince et al. [42], sausage spoilage	Predict sausage spoilage intensity under limited observations.	TVAE augmentation improved prediction, while SHAP/LIME supported interpretation.	Tabular variational autoencoder (TVAE) augmentation plus explainable ML.	Experimental/reference method
B1. Pathogens, antimicrobial resistance, and spoilage	Ince et al. [43], VOC-based sausage spoilage	Predict spoilage from volatile-organic-compound profiles.	TVAE augmentation supported VOC-based spoilage prediction.	Tabular variational autoencoder (TVAE) augmentation plus explainable ML.	Experimental/reference method
B1. Pathogens, antimicrobial resistance, and spoilage	Meenakshi et al. [33], Listeria in Queso fresco	Detect Listeria using NIR spectral analysis.	GAN-augmented spectra improved detection in a food matrix.	GAN plus NIR analysis.	Experimental/reference method
B1. Pathogens, antimicrobial resistance, and spoilage	Li, Q. et al. [49], single-cell pathogens	Identify foodborne pathogens from Raman evidence.	GAN-enhanced Raman spectrograms improved single-cell classification.	Raman Mel spectrograms plus GAN.	Experimental/reference method
B1. Pathogens, antimicrobial resistance, and spoilage	You et al. [50], Salmonella antimicrobial resistance	Build a predictive platform for Salmonella resistance.	LLM and quantum-computing evidence supported knowledge-assisted prediction.	Large language model plus quantum computing.	Dataset/model-only
B1. Pathogens, antimicrobial resistance, and spoilage	Sun et al. [34], limited-data pathogen identification	Identify foodborne pathogens from small Raman datasets.	Peak-aware attention improved limited-data identification.	Raman attention model with synthetic-data support.	Experimental/reference method
B2. Mycotoxins, chemical contaminants, and residues	Hao et al. [51], PAHs in roasted lamb	Analyze polycyclic aromatic hydrocarbons from spectral and chemical evidence.	Data-enhanced learning improved contaminant estimation.	Machine learning with generative/data augmentation.	Experimental/reference method
B2. Mycotoxins, chemical contaminants, and residues	Williams et al. [40], aflatoxin in pistachios	Estimate aflatoxin infection from hyperspectral images.	Data-enhanced hyperspectral learning supported contamination-level estimation.	Hyperspectral imaging plus machine learning.	Experimental/reference method
B2. Mycotoxins, chemical contaminants, and residues	Sebti et al. [46], ochratoxin A in grapes	Classify ochratoxin contamination in real time.	AI-enhanced sensing supported rapid contamination screening.	IoT sensing with data enhancement.	Experimental/reference method
B2. Mycotoxins, chemical contaminants, and residues	Wang et al. [44], Aflatoxin M1 prediction	Predict Aflatoxin M1 under distributed data conditions.	Federated learning and data enhancement improved prediction.	Federated learning with data enhancement.	Experimental/reference method
B2. Mycotoxins, chemical contaminants, and residues	Wang et al. [45], spatiotemporal Aflatoxin M1 risk	Predict raw-milk Aflatoxin M1 over space and time.	Spatiotemporal attention improved risk forecasting.	STANet and synthetic/data-enhanced evidence.	Experimental/reference method
B2. Mycotoxins, chemical contaminants, and residues	Zhu et al. [52], spectral aflatoxin B1 prediction	Predict pixel-level Aflatoxin B1 content from spectral data.	Fine-tuned LLM improved contaminant prediction.	Fine-tuned large language model.	Experimental/reference method
B2. Mycotoxins, chemical contaminants, and residues	Zhang et al. [32], small-molecule toxins	Identify toxins through ultraviolet resonance Raman evidence.	Frequency-consistent augmentation improved toxin identification.	Generative Raman augmentation.	Experimental/reference method
B3. Authenticity and adulteration	Bao et al. [29], camel milk adulteration	Generative and explainable NIR adulteration detection.	Improved discrimination under limited spectral evidence.	U-Mamba-Spectra; generative NIR learning.	Experimental/reference method
B3. Authenticity and adulteration	He et al. [30], sweetener adulteration	Detect synergistic sweetener adulteration using Raman spectroscopy.	CVAE augmentation improved discrimination and feature robustness.	ResAttnCVAE and gated SpecFormer.	Experimental/reference method
B3. Authenticity and adulteration	Yang et al. [31], medicine–food homologous herbs	Identify medicine–food homologous herbs from NIR spectra.	NIR-GAN augmentation improved small-sample identification.	NIR-GAN.	Experimental/reference method
B3. Authenticity and adulteration	Shen et al. [26], salted-goose breed discrimination	Discriminate salted-goose breeds using sensory and instrumental evidence.	GAN-integrated learning improved breed authentication.	Chemometrics, sensory evaluation, GAN framework.	Human/sensory/expert-informed
B2. Mycotoxins, chemical contaminants, and residues	Zhao et al. [38], apple pesticide residues	Detect apple pesticide residues from FT-NIR spectra.	GAN reconstruction and reverse feature fusion improved detection.	CNN-BiGRU-self-attention plus GAN reconstruction.	Experimental/reference method
B2. Mycotoxins, chemical contaminants, and residues	Tan et al. [39], Hami-melon pesticide residues	Identify pesticide residues from hyperspectral images.	DCGAN augmentation improved residue classification.	Improved DCGAN plus hyperspectral imaging.	Experimental/reference method
B4. Safety-risk classification in complex environments	Du et al. [53], mushroom identification	Generate complex-environment mushroom images for food-safety identification.	Synthetic augmentation improved identification under class and scene variability.	Diffusion/synthetic-image augmentation.	Dataset/model-only
B5. Safety text analytics and surveillance	Xiong et al. [54], food-safety review classification	Classify food-safety reviews using label-aware LLMs.	LLM and label embedding improved text classification.	Large language model plus label embedding.	Dataset/model-only

## Appendix C. T3 Quality, Nondestructive Inspection, and Property Prediction

Table A3 provides paper-level traceability for Theme T3.

**Table A3:** Paper-level traceability for T3. Theme evidence appearances: *n* = 9.

Subtheme	Study	Study Focus	Principal Finding	Method or Mechanism	Reported Validation
C1. Synthetic spectra for property prediction	Zhang et al. [57], fruit spectral generation	Generate hundreds of wavelengths from limited multispectral inputs.	Generated spectra supported downstream fruit-quality applications.	Deep spectral generation.	Experimental/reference method
C1. Synthetic spectra for property prediction	Li, X. et al. [35], watermelon soluble solids	Predict soluble-solids content from NIR spectra.	Auxiliary regression GAN augmentation improved quantitative prediction.	Regression GAN plus chemometrics.	Experimental/reference method
C2. Reconstruction and diffusion-based imaging	Bonettini et al. [36], hyperspectral X-ray denoising	Denoise hyperspectral X-ray measurements for food inspection.	Autoencoder reconstruction improved usable signal quality in real time.	Autoencoder variants.	Dataset/model-only
C2. Reconstruction and diffusion-based imaging	Yi et al. [37], LF-NMR fruit imaging	Reconstruct low-field NMR images for nondestructive testing.	DDPM super-resolution improved image usability.	Denoising diffusion probabilistic model.	Experimental/reference method
C2. Reconstruction and diffusion-based imaging	Chen et al. [27], small-data food image generation	Generate single-category food images under small-data conditions.	The improved diffusion model increased visual diversity and classifier support.	Improved diffusion model.	Dataset/model-only
C2. Reconstruction and diffusion-based imaging	Li, B. et al. [41], pumpkin-seed plumpness	Detect pumpkin-seed plumpness from terahertz images.	Compressed sensing and super-resolution improved internal-quality visibility.	Terahertz compressed sensing plus Real-ESRGAN super-resolution.	Experimental/reference method
C3. Zero-shot and multimodal quality interpretation	Zhou, P. et al. [28], zero-shot food detection	Synthesize knowledge-enhanced visual features for unseen food classes.	Feature synthesis improved real-world zero-shot detection.	Region-feature diffusion model for knowledge-enhanced feature synthesis.	Dataset/model-only
C3. Zero-shot and multimodal quality interpretation	Wu, H. et al. [58], temporal fruit classification	Fuse vision and spectral information for zero-shot fruit classification.	LLM-led fusion improved temporal transfer.	LLM-led vision-spectral fusion.	Dataset/model-only
C3. Zero-shot and multimodal quality interpretation	Yin, X.-Y. et al. [59], image-based food quality	Analyze food quality through LLM-driven image interpretation.	The framework improved cross-modal quality interpretation.	Large language and vision-language models.	Dataset/model-only

## Appendix D. T4 Sensory, Formulation, Flavor, and Process Innovation

Table A4 provides paper-level traceability for Theme T4.

**Table A4:** Paper-level traceability for T4. Theme evidence appearances: *n* = 8.

Subtheme	Study	Study Focus	Principal Finding	Method or Mechanism	Reported Validation
D1. Recipe, formulation, and process-data support	Tkachenko et al. [60], energy bar recipes	Generate and compare energy bar formulations.	LLM-generated recipes were prepared and evaluated for sensory quality and storage stability.	ChatGPT (GPT-4o), Gemini 1.5 Pro, and Claude 3.5 Sonnet.	Human/sensory/expert-informed
D1. Recipe, formulation, and process-data support	Bölücü et al. [61], food-extrusion data supplementation	Use LLMs to supplement a food-extrusion dataset.	Generated records broadened early process-data exploration but remained unverified experimentally.	Large language models.	Dataset/model-only
D2. Sensory description and chemosensory creativity	Torrico [62], brownie sensory evaluation	Assess ChatGPT as a sensory evaluator.	Generated evaluations showed partial alignment with human sensory judgment.	ChatGPT case study.	Human/sensory/expert-informed
D2. Sensory description and chemosensory creativity	Wang and Pellegrino [63], chemosensory creativity	Automate assessment of chemosensory creativity.	LLM ratings approximated parts of human judgment.	Large language models.	Human/sensory/expert-informed
D3. Astringency, taste, texture, and olfactory reasoning	He et al. [64], astringency knowledge and prediction	Integrate astringency databases, prediction, and antibacterial reasoning.	Astringency predictions were validated using electronic-tongue measurements.	Large language models, machine learning, and electronic-tongue validation.	Experimental/reference method
D3. Astringency, taste, texture, and olfactory reasoning	Lee et al. [65], coffee electronic nose	Integrate E-nose sensing, LLMs, and knowledge graphs.	The integrated system improved olfactory reasoning and interpretability.	E-nose, LLM, and knowledge graph.	Human/sensory/expert-informed
D3. Astringency, taste, texture, and olfactory reasoning	Ma et al. [66], peptide, taste, and texture	Transfer foundation models across molecular taste and visual texture tasks.	Foundation models integrated heterogeneous food signals.	Foundation models and transfer learning.	Dataset/model-only
D4. Synthetic sensory data prediction	Glumac et al. [47], smoothie sensory perception	Predict sensory attributes in texture-modified plant-based smoothies.	Literature-informed synthetic data supported prediction of simulated sensory attributes.	Synthetic data plus deep neural networks.	Dataset/model-only

## Appendix E. T5 Foundation-Model and Multimodal Decision Support

Table A5 provides paper-level traceability for Theme T5.

**Table A5:** Paper-level traceability for T5. Theme evidence appearances: *n* = 13.

Subtheme	Study	Study Focus	Principal Finding	Method or Mechanism	Reported Validation
E1. Food-safety text and knowledge systems	Xiong et al. [54], food-safety review classification	Classify food-safety reviews using label-aware LLMs.	LLM and label embedding improved text classification.	Large language model plus label embedding.	Dataset/model-only
E1. Food-safety text and knowledge systems	You et al. [50], Salmonella antimicrobial resistance	Build a predictive platform for Salmonella resistance.	LLM and quantum-computing evidence supported knowledge-assisted prediction.	Large language model plus quantum computing.	Dataset/model-only
E1. Food-safety text and knowledge systems	Zhu et al. [52], spectral aflatoxin B1 prediction	Predict pixel-level Aflatoxin B1 content from spectral data.	Fine-tuned LLM improved contaminant prediction.	Fine-tuned large language model.	Experimental/reference method
E2. Quality and multimodal interpretation	Ma et al. [69], high-throughput nutrition screening	Use vision-language models for nutrition screening.	Cross-modal transfer supported high-throughput interpretation.	Vision-language foundation model.	Dataset/model-only
E2. Quality and multimodal interpretation	Wu, H. et al. [58], temporal fruit classification	Fuse vision and spectral information for zero-shot fruit classification.	LLM-led fusion improved temporal transfer.	LLM-led vision–spectral fusion.	Dataset/model-only
E2. Quality and multimodal interpretation	Yin, X.-Y. et al. [59], image-based food quality	Analyze food quality through LLM-driven image interpretation.	The framework improved cross-modal quality interpretation.	Large language and vision-language models.	Dataset/model-only
E3. Sensory, flavor, and olfactory reasoning	He et al. [64], astringency knowledge and prediction	Integrate astringency databases, prediction, and antibacterial reasoning.	Astringency predictions were validated using electronic-tongue measurements.	Large language models, machine learning, and electronic-tongue validation.	Experimental/reference method
E3. Sensory, flavor, and olfactory reasoning	Lee et al. [65], coffee electronic nose	Integrate E-nose sensing, LLMs, and knowledge graphs.	The integrated system improved olfactory reasoning and interpretability.	E-nose, LLM, and knowledge graph.	Human/sensory/expert-informed
E3. Sensory, flavor, and olfactory reasoning	Ma et al. [66], peptide, taste, and texture	Transfer foundation models across molecular taste and visual texture tasks.	Foundation models integrated heterogeneous food signals.	Foundation models and transfer learning.	Dataset/model-only
E3. Sensory, flavor, and olfactory reasoning	Torrico [62], brownie sensory evaluation	Assess ChatGPT as a sensory evaluator.	Generated evaluations showed partial alignment with human sensory judgment.	ChatGPT case study.	Human/sensory/expert-informed
E3. Sensory, flavor, and olfactory reasoning	Wang and Pellegrino [63], chemosensory creativity	Automate assessment of chemosensory creativity.	LLM ratings approximated parts of human judgment.	Large language models.	Human/sensory/expert-informed
E4. Formulation and process-data support	Bölücü et al. [61], food-extrusion data supplementation	Use LLMs to supplement a food-extrusion dataset.	Generated records broadened early process-data exploration but remained unverified experimentally.	Large language models.	Dataset/model-only
E4. Formulation and process-data support	Tkachenko et al. [60], energy bar recipes	Generate and compare energy bar formulations.	LLM-generated recipes were prepared and evaluated for sensory quality and storage stability.	ChatGPT (GPT-4o), Gemini 1.5 Pro, and Claude 3.5 Sonnet.	Human/sensory/expert-informed

## Appendix F. Search Strategy and Report-Level Exclusions

### Appendix F.1. Exact Scopus Search Strategy

Database: Scopus. Search date: 13 June 2026. Records returned: 438. The query includes publication-year, English-language, and article/review document-type filters. The 11 July 2026 date refers to the subsequent full-text availability and eligibility check.

TITLE-ABS-KEY (
    (
      food* W/10 (
        product* OR formulation* OR design OR technolog* OR quality OR safety OR
        analys* OR process* OR engineering OR composition OR propert* OR matrix OR
        authenticity OR authenticat* OR adulterat* OR fraud OR contamination OR
        microbiolog* OR pathogen* OR spoilage OR “shelf life” OR “shelf-life” OR
        sensory OR flavor* OR flavour* OR aroma* OR taste* OR texture* OR
        nutrition* OR ingredient* OR recipe* OR image* OR classification OR prediction OR
        detection OR spectroscopy OR hyperspectral OR “near infrared” OR NIR
      )
      OR
      “functional food*” OR “novel food*” OR “foodborne” OR “food packaging” OR
      “food product development” OR “new food product development” OR
      “food quality” OR “food safety” OR “food analysis” OR “food processing” OR
      “food formulation” OR “food design” OR “food authenticity” OR
      “food adulteration” OR “food fraud” OR “food composition” OR
      “food property prediction” OR “food composition prediction” OR
      “food image*” OR “food classification” OR “food recognition”
    )
    AND
    (
      “generative artificial intelligence” OR “generative AI” OR GenAI OR
      “large language model*” OR “large-language model*” OR LLM OR LLMs OR
      ChatGPT OR “generative pre-trained transformer*” OR
      “generative pretrained transformer*” OR GPT OR “GPT-3” OR “GPT-4” OR “GPT-4o” OR
      “foundation model*” OR “vision-language model*” OR VLM OR VLMs OR
      “large multimodal model*” OR LMM OR LMMs OR “multimodal model*” OR
      “deep generative model*” OR “generative model*” OR
      “generative adversarial network*” OR GAN OR GANs OR
      “conditional generative adversarial network*” OR “conditional GAN” OR cGAN OR cGANs OR
      “cycle-consistent generative adversarial network*” OR CycleGAN OR
      “Wasserstein generative adversarial network*” OR “Wasserstein GAN” OR WGAN OR
      “variational autoencoder*” OR VAE OR VAEs OR
      “conditional variational autoencoder*” OR CVAE OR CVAEs OR
      “diffusion model*” OR “denoising diffusion model*” OR
      “denoising diffusion probabilistic model*” OR DDPM OR
      “normalizing flow*” OR
      “synthetic data” OR “synthetic image*” OR “synthetic dataset*” OR
      “synthetic sample*” OR “synthetic generation”
    )
    AND
    (
      “product development” OR “new product development” OR formulation OR
      “recipe generation” OR “recipe design” OR “ingredient optimization” OR
      “quality prediction” OR “quality assessment” OR “quality evaluation” OR
      “quality control” OR prediction OR classification OR detection OR
      “safety monitoring” OR “safety assessment” OR
      “contaminant detection” OR “pathogen detection” OR “microbial detection” OR
      “adulteration detection” OR “authenticity assessment” OR “fraud detection” OR
      “defect detection” OR “spoilage detection” OR “sensory prediction” OR
      “sensory evaluation” OR “flavor prediction” OR “flavour prediction” OR
      “aroma prediction” OR “taste prediction” OR “texture prediction” OR
      “shelf life” OR “shelf-life” OR “nutrient prediction” OR
      “nutritional profiling” OR “process optimization” OR
      “food property prediction” OR “food composition prediction” OR
      validation OR “human-in-the-loop” OR “human in the loop”
    )
  )
  AND PUBYEAR > 2015
  AND PUBYEAR < 2027
  AND ( LIMIT-TO ( LANGUAGE , “English” ) )
  AND ( LIMIT-TO ( DOCTYPE , “ar” ) OR LIMIT-TO ( DOCTYPE , “re” ) )

### Appendix F.2. Reports Excluded After Full-Text Assessment

Specifically, 6 of the 71 assessed full-text reports were excluded for the reasons below. The two reports that could not be retrieved are counted separately in Figure 1 and are not represented as assessed full-text exclusions.

Table A6 lists the six reports excluded after full-text assessment and the corresponding eligibility criterion not met.

**Table A6:** Report-level full-text exclusions and the eligibility criterion not met.

Record ID	Report and Identifier	Reason for Exclusion
389	Generative Artificial Intelligence for Synthetic Spectral Data Augmentation in Sensor-Based Plastic Recycling [112].	The application concerns plastic recycling rather than a material food-product or food-packaging decision within the review scope.
197	Emerging analytical techniques for lipid profiling in food products: insights into processing effects and quality control [113].	Food relevance is present, but an eligible generative, foundation-model, or explicit synthetic data mechanism is not central to the contribution.
90	Application of Linear Mixed Model in Longitudinal Study to Optimize Effect of Ultrasound on Color of Minimally Processed Granny Smith Apple [114].	The analytical contribution uses conventional statistical modeling rather than an eligible generative or foundation-model mechanism.
106	Deep eutectic solvent and improved needle filter-based liquid-membrane microextraction combined with high performance liquid chromatography for the determination of sulfonamide and fluoroquinolone residues in milk [115].	The study concerns analytical sample preparation and chromatography without an eligible AI mechanism.
331	An approach towards IoT-based predictive service for early detection of diseases in poultry chickens [116].	The main decision concerns animal-health prediction rather than the required food-product analytical application; an eligible central generative mechanism is not established.
147	Product Helpfulness Detection With Novel Transformer Based BERT Embedding and Class Probability Features [117].	The contribution concerns generic product-review helpfulness. Use of a food-labeled review dataset does not establish the required food-safety, authenticity, quality, or related analytical decision.

## Appendix G. Scope and Search-String Sensitivity

Table A7 reports exploratory comparisons using the original publication-level categories. The title-domain subset excludes all 10 sensory/formulation/process and packaging/nutrition contributions listed in Table A8. The narrowest subset also excludes Chen et al. [27], food-image generation, and Zhou, P. et al. [28], zero-shot food detection. These within-corpus restrictions do not recode individual publications or alter the retained corpus. The matched Scopus reruns reported below provide a separate test of search-string sensitivity.

**Table A7:** Reported validation counts under narrower application scopes.

Comparison Set	Count	Dataset/Model	Experimental/Reference	Human/Expert	Industrial/Field
Full direct core	41	13	23	5	0
Title-domain reports	31	8	22	1	0
Title-domain, without two image-only demonstrations	29	6	22	1	0
Adjacent domains (complement of 31)	10	5	1	4	0

The corresponding primary-role counts (GAN/VAE; foundation/LLM/multimodal; diffusion/super-resolution; other synthetic data) are 19/13/5/4 for 41 reports, 18/5/5/3 for 31 reports, and 18/5/3/3 for 29 reports. The narrower scopes retain the zero industrial count but alter validation proportions. For example, dataset/model-only evidence falls from 13/41 (31.7%) to 8/31 (25.8%) and 6/29 (20.7%). These are scope-dependent descriptions, not estimates of prevalence or quality.

Excluding the supporting review by Mann et al. [20] from the bibliographic profile changes the total from 65 to 64 and the 2026 count from 26 to 25. The other year counts remain 2 (2022), 4 (2023), 5 (2024), and 28 (2025). Thus, 2025–2026 share changes from 54/65 (83.1%) to 53/64 (82.8%); the 41-paper analytical counts and leading source totals are unaffected.

**Table A8:** The 10 adjacent-domain reports within the direct core.

Publication	Adjacent Application	Recorded Validation
Ma et al. [69]	Integrating Vision-Language Models for Accelerated High-Throughput Nutrition Screening	Dataset/model-only
Lee et al. [65]	Enhancing Artificial Olfactory Reasoning via Integration of Electronic Nose Sensing, Large Language Models, and Knowledge Graphs: A Case Study on Coffee E-Nose	Human/sensory/expert-informed
He et al. [64]	Database, prediction, and antibacterial research of astringency based on large language models	Experimental/reference method
Bölücü et al. [61]	An Evaluation of Large Language Models for Supplementing a Food Extrusion Dataset	Dataset/model-only
Liu et al. [48]	Sensor-Integrated Inverse Design of Sustainable Food Packaging Materials via Generative Adversarial Networks	Dataset/model-only
Wang and Pellegrino [63]	Automating chemosensory creativity assessment with large language models	Human/sensory/expert-informed
Ma et al. [66]	Leveraging foundation models and transfer learning for peptide transport prediction, molecular taste classification, and visual texture analysis	Dataset/model-only
Torrico [62]	The Potential Use of ChatGPT as a Sensory Evaluator of Chocolate Brownies: A Brief Case Study	Human/sensory/expert-informed
Glumac et al. [47]	Synthetic data and deep neural networks enable prediction of sensory perception attributes in texture-modified plant-based smoothies	Dataset/model-only
Tkachenko et al. [60]	Determining the prospects of using artificial intelligence for generating energy bar recipes	Human/sensory/expert-informed

These 10 reports comprise 8 sensory/formulation/flavor/process applications and 2 packaging/nutrition applications. Six are assigned to dataset/model evaluation and four to human/sensory/expert assessment. They are members of the direct core, not the separate 24-publication supporting tier.

The matched search-string comparison on 10 September 2026 returned 521 records for the original query, 960 for the relaxed variant, and 96 for the tightened variant. The sets were completely nested: tightened within original, and original within relaxed. The original and relaxed sets each recovered 65/65 retained publications (41/41 direct and 24/24 supporting); the tightened set recovered 22/65 (16/41 direct and 6/24 supporting). Relative to the same-date original rerun, relaxation added 439 records (84.3%), while tightening removed 425 (81.6%) and omitted 43 known included publications. These figures describe retrieval breadth and known-set recovery, not search precision or exhaustive recall. A staged conservative assessment of the 439 relaxed-only records applied the original scope and 13 June evidence cut-off. At title/abstract stage, 391 records were clearly excluded, and 15 borderline records were conservatively not advanced. Thirty-three records remained sufficiently relevant for closer assessment; five of these were published after the evidence cut-off. Detailed assessment of the 28 pre-cutoff candidates excluded 8, leaving 20 pre-cutoff sensitivity signals: 5 most compatible with the direct-core rules and 15 with supporting/context relevance. Thus, 419/439 records (95.4%) did not remain in the pre-cut-off relevant set under this diagnostic. Mapping the five direct-core-compatible candidates to the existing categories would preserve all five themes and the zero industrial/field count, while changing experimental/reference validation from 23 to 27, dataset/model validation from 13 to 13, human/sensory/expert validation from 5 to 6, and the title-aligned share from 75.6% to 73.9%. This impact mapping is a robustness diagnostic rather than a selective corpus update. Figure 1 therefore retains the actual 438-record identification search of 13 June 2026, and the primary 65-publication synthesis remains the evidence set generated by that predefined search and independent dual-review process.

For reproducibility, the relaxed variant deletes only the third AND application block from the exact query in Appendix F.1. The tightened variant retains the food-context and model blocks unchanged and replaces that application block with the expression below. Both variants retain PUBYEAR > 2015, PUBYEAR < 2027, English, and article/review limits. The executed strategies are nested variants, not separate eligibility criteria.

(“quality prediction” OR “quality assessment” OR “quality evaluation” OR “quality control” OR “safety monitoring” OR “safety assessment” OR “contaminant detection” OR “pathogen detection” OR “microbial detection” OR “adulteration detection” OR “authenticity assessment” OR “fraud detection” OR “defect detection” OR “spoilage detection” OR “food property prediction” OR “food composition prediction”)

## Appendix H. Illustrative Gate-to-Evidence Mapping

Table A9 connects examples from the retained corpus to the questions operationalized in Table 14. It distinguishes reported supporting evidence from requirements that remain unestablished. The entries are explanatory illustrations rather than study-quality scores, legal compliance determinations, or binary gate ratings.

**Table A9:** Corpus examples and limits of gate-level interpretation.

Gate	Authentic Corpus Illustration	Evidence Boundary
1. Provenance	Du et al. [2], Raman pathogen spectra; Ince et al. [42], sausage spoilage augmentation.	The source and augmentation contexts illustrate the required lineage checks. Complete partition, ownership, and contamination assessments are additional criteria for a task-specific provenance evaluation.
2. Technical validity	Yi et al. [37], LF-NMR reconstruction; Chen et al. [27], small-dataset food-image generation.	Technical comparisons demonstrate particular capabilities. Dataset-level image generation does not establish an independently measured safety or quality outcome.
3. Food-science validity	Meenakshi et al. [33], Listeria in Queso fresco; Chen et al. [27], food-image generation.	The review records reference-linked evidence in the former and dataset/model evidence in the latter. That contrast is not a completed official-method equivalence test or proof of failure by the latter study.
4. Human calibration	Shen et al. [26], sensory/instrumental authenticity assessment; Tkachenko et al. [60], energy bar prototypes; Bölücü et al. [61], extrusion data.	Human or expert evidence in the first two contrasts with dataset/model evidence in the third. None of those category labels alone certifies a standards-qualified panel or safe decision workflow.
5. Deployment governance	The 41-report direct core contains no qualifying prospective industrial report.	The corpus supplies no demonstrated complete Gate 5 case. Operational performance, legal eligibility, monitoring, accountability, and change control require prospective workflow evaluation.
